# Deep whole-genome sequencing of 90 Han Chinese genomes

**DOI:** 10.1093/gigascience/gix067

**Published:** 2017-07-31

**Authors:** Tianming Lan, Haoxiang Lin, Wenjuan Zhu, Tellier Christian Asker Melchior Laurent, Mengcheng Yang, Xin Liu, Jun Wang, Jian Wang, Huanming Yang, Xun Xu, Xiaosen Guo

**Affiliations:** 1 BGI-Shenzhen, Build 11, Beishan Industrial Zone, Yantian District, Shenzhen, 518083, China; 2 BGI Genomics, BGI-Shenzhen, Building NO. 7, BGI Park, No. 21 Hongan 3rd Street, Yantian District, Shenzhen, 518083, China; 3 Department of Biology, University of Copenhagen, Nørregade 10, PO Box 2177 1017 Copenhagen, Denmark; 4 James D. Watson Institute of Genome Sciences, 866 Yuhangtang Road, Hangzhou, Zhejiang Province, 310058, P. R. China; 5 Shenzhen Key Laboratory of Neurogenomics, BGI-Shenzhen, Build 11, Beishan Industrial Zone, Yantian District, Shenzhen, 518083, China

**Keywords:** high-coverage whole-genome sequencing, Han Chinese genomes, *de novo* assembly, genetic variations

## Abstract

Next-generation sequencing provides a high-resolution insight into human genetic information. However, the focus of previous studies has primarily been on low-coverage data due to the high cost of sequencing. Although the 1000 Genomes Project and the Haplotype Reference Consortium have both provided powerful reference panels for imputation, low-frequency and novel variants remain difficult to discover and call with accuracy on the basis of low-coverage data. Deep sequencing provides an optimal solution for the problem of these low-frequency and novel variants. Although whole-exome sequencing is also a viable choice for exome regions, it cannot account for noncoding regions, sometimes resulting in the absence of important, causal variants. For Han Chinese populations, the majority of variants have been discovered based upon low-coverage data from the 1000 Genomes Project. However, high-coverage, whole-genome sequencing data are limited for any population, and a large amount of low-frequency, population-specific variants remain uncharacterized. We have performed whole-genome sequencing at a high depth (∼×80) of 90 unrelated individuals of Chinese ancestry, collected from the 1000 Genomes Project samples, including 45 Northern Han Chinese and 45 Southern Han Chinese samples. Eighty-three of these 90 have been sequenced by the 1000 Genomes Project. We have identified 12 568 804 single nucleotide polymorphisms, 2 074 210 short InDels, and 26 142 structural variations from these 90 samples. Compared to the Han Chinese data from the 1000 Genomes Project, we have found 7 000 629 novel variants with low frequency (defined as minor allele frequency < 5%), including 5 813 503 single nucleotide polymorphisms, 1 169 199 InDels, and 17 927 structural variants. Using deep sequencing data, we have built a greatly expanded spectrum of genetic variation for the Han Chinese genome. Compared to the 1000 Genomes Project, these Han Chinese deep sequencing data enhance the characterization of a large number of low-frequency, novel variants. This will be a valuable resource for promoting Chinese genetics research and medical development. Additionally, it will provide a valuable supplement to the 1000 Genomes Project, as well as to other human genome projects.

## Data Description

### Background

Next-generation sequencing has become widely utilized in human genetics research compared to previous technologies, in particular for genome-wide association studies (GWAS). The 1000 Genomes Project (1000GP) has distributed a standard pattern of more than 88 million variants, providing for research use a global genetic reference panel [1–3]. The Haplotype Reference Consortium (HRC) has also constructed a distinct human reference panel, consisting of 39 235 157 single nucleotide polymorphisms (SNPs) [4]. However, most of the samples contributed to either the 1000GP or to the HRC have an average sequencing depth of only ×4∼8, which makes characterization of low-frequency variants (minor allele frequency [MAF] < 5%), and especially rare variants (MAF < 1%) [5], difficult. These 2 projects therefore cannot supply a high-resolution spectrum of variations for many human populations, in particular for Han Chinese [6].

Low-coverage sequencing can be used to generate a high-quality variation set supplemented by imputation, though imputation will perform poorly in correctly calling rare variants, using the current set of typed markers and reference panels [6, 7]. Whole-exome sequencing (WES) is a viable method to elucidate associations between rare variants and human diseases, when these are to be found in coding regions; however, WES cannot characterize non-coding regions, which encompass 98% of the human genome and are increasingly recognized to play an important role in some human traits [8, 9]. The 1000GP, the HRC and other human genome projects have generated extensive human variation catalogues, which can be used to design high-density genotyping arrays. However, these chips are likely to miss rare or low-frequency alleles [9]. Furthermore, for Han Chinese genomes, a large number of population-specific variants can be expected to be absent from the imputation variant panel set of 1000GP. Therefore, it is necessary to further characterize the Han genome using high-depth whole-genome sequencing.

Besides SNPs and InDels, structural variants (SVs) have also been found in recent studies [10, 11] to be associated with human diseases that contribute to human genetic diversity; 68 818 SVs have already been detected using the data of the 1000GP, and an integrated SV map for global human population has been generated [12]. However, SV identification using short reads from low-coverage sequencing data remains challenging [13]. Assembly-based SV calling based on high-coverage sequencing data provides a feasible and powerful method [14].

In this study, we have sequenced the genomes of 90 Han Chinese samples extracted from 1000GP, at an average sequencing depth of ∼×80, including 7 hitherto un-sequenced samples from 1000GP. We have built a high-resolution spectrum of genetic variation for the Han Chinese population, based on high-coverage genomic data, including SNPs, InDels, and SVs. These data provide a valuable resource for performing further genomics/genetics studies in Han Chinese populations, especially for exploring the functional roles of these low-frequency novel variants. Additionally, as a valuable supplement, the data will enrich genetic variation catalogues of global human populations.

### Samples

Genomic DNA was extracted from the cell lines of 90 unrelated Chinese samples from the 1000GP, currently deposited at Coriell Institute for Medical Research. Forty-five of these 90 samples were taken from Southern Han Chinese (CHS) individuals, and the remaining 45 were taken from Han Chinese individuals from Beijing (CHB). Of these samples, seven were hitherto unsequenced by the1000GP, including 5 CHB and 2 CHS.

### Ethics Statement

All samples used in this study were obtained from the 1000 Genomes Project cell line collection at the Coriell cell line repository and were qualified by the ethics protocols of the 1000GP. According to the statement of informed consent, all samples held by the 1000GP, including these 90 Chinese individuals, have been released and shared to the public. All individuals have consented to allowing their genomic data being used in the analyses of the project and being freely distributed for future research. Public distribution of the sequencing data and genotypes has also been explicitly consented to. This study has been also approved by the Institutional Review Board on Bioethics and Biosafety (reference number: BGI-IRB 16 101).

### Sequencing

Illumina HiSeq 2000 Paired Library preparation was done in accordance with the manufacturer's instructions (Illumina, San Diego, CA, USA). We performed cluster generation using the Illumina cluster station, with workflow as follows: template hybridization, isothermal amplification, linearization, blocking, denaturation, and sequencing primer hybridization. Fluorescent images were processed to sequences using the standard Illumina base-calling pipeline. We built 5 ranks of DNA libraries with different insert sizes (170 bp, 500 bp, 2 kb, 5 kb, 10 kb, 20 kb) (Table 1). The average depth of CHS was 71.87 ± 23.52, and that of CHB was 82.36 ± 14.13. The average genome coverage of CHS was 99.65% ± 0.34%, and that of CHB was 99.60% ± 0.30% (Table 2).

**Table 1:** The sequencing depth of different library insert sizes

Library insert size	Sequencing depth (fold)	Standard deviation
180 bp	51.78	8.11
500 bp	12.74	2.54
2000 bp	5.01	1.08
5000 bp	5.02	2.08
10 000 bp	5.62	2.22
20 000 bp	6.68	2.52
<1000 bp	64.52	8.11
>1000 bp	22.33	3.90
Total	86.85	8.53

Sequencing depth is calculated as total sequencing base/3e10.

**Table 2:** Deep whole-genome sequencing data of 90 Chinese samples

	CHS	CHB	Total
Number of individuals	45	45	90
Raw bases (Gb)	231.61 ± 72.61	264.24 ± 44.92	247.69 ± 56.54
Mapped bases (Gb)	212.35 ± 68.96	243.28 ± 41.81	227.57 ± 53.74
Average sequencing depth (fold)	71.87 ± 23.52	82.36 ± 14.13	77.02 ± 18.37

### SNP/INDEL discovery

Read alignment was performed using the *aln* algorithm of BWA 0.6.1 (BWA, RRID:SCR_010910) [15], using reads with insert sizes ranging from 180 bp to 500 bp, to align against the human genome (hg19/GRCh37). We used Genome Analysis Tool Kit (v. 2.7.1; GATK, RRID:SCR_001876) [16, 17] to remove duplications, realign around InDels, and recalibrate alignment quality scores. SNPs and InDels calling was performed using GATK UnifiedGenotyper. After Variant Quality Score Recalibration filtering, we ultimately obtained 12 568 804 SNPs and 2 074 210 InDels. The step-by-step procedures and command lines of the variant calling procedures have been saved in detail on the protocols.io platform [18]. Of these variants, 12 536 571 SNPs and 1 472 925 InDels were bi-allelic, 4 573 367 were rare variants (MAF ≤ 1%), 3 013 158 were low frequency (1% < MAF ≤ 5%), and 6 422 971 were common (MAF > 5%). Gene-based annotations for SNPs and InDels were also performed using ANNOVAR (ANNOVAR, RRID:SCR_012821) [19] based on the reference genome of Hg19. We found that 5 651 762 SNPs and 982 480 InDels were distributed in gene regions, respectively, and that more than half of the variants were found in the intergenic regions (Table 3).

**Table 3:** Gene-based annotation of SNPs and InDels

Regions	SNPs	InDels
Intron	5 072 778	889 195
CDS	127 027	5916
5^΄^UTRs	15 823	1754
3^΄^UTRs	90 167	18 062
Upstream	174 016	33 074
Downstream	171 951	34 479
Intergenic	6 917 042	1 091 730
Total variant	12 568 804	2 074 210

### Variation evaluation

To evaluate the SNP set, we first randomly selected 22 samples and performed genotyping using the Illumina Infinium OmniZhongHua-8 v1 BeadChip; 407 040 ± 2635 SNPs were obtained, and the concordance rate was 99.94% ± 0.02% (false discovery rate [FDR] = 0.06% ± 0.02%). We then compared our SNP set with several public genotype data sets, including Affymetrix Affy 6.0, Illumina Omni 2.5 arrays, and the variation set of the 1000GP (phase III). For Affymetrix Affy 6.0 and Illumina Omni 2.5 arrays, we compared the variants of 86 individuals with genotyping overlap between the 2 platforms. As described in the comparison with Illumina Infinium OmniZhongHua-8 v. 1 BeadChip array, FDR rates were both below 0.1% (Table 4). Finally, we ran a comparison between our SNP set and the 1000GP (phase III) SNP set and counted the SNP set overlap between these 2 sets, enumerating the FDR to be 0.32% ± 0.07%. For InDels, we also compared 2 sets using the same method, for which the FDR was 2.72% ± 0.15% (Table 4).

**Table 4:** Validations results of SNPs and InDels

Types	Referenced variation set	Sample size	Total sites	Concordance sites	Concordance rate	FDR
SNP	Illumina Infinium OmniZhongHua-8	22	407 040 ± 2635	406 790 ± 2674	99.94% ± 0.02%	0.06% ± 0.02%
	Affymetrix Affy 6.0	86	406 354 ± 2064	406 011 ± 2109	99.92% ± 0.02%	0.08% ± 0.02%
	Illumina Omni 2.5	86	678 718 ± 2783	678 253 ± 2805	99.93% ± 0.01%	0.07% ± 0.01%
	1KG phase III	83	10 678 197 ± 892	10 648 954 ± 7813	99.68% ± 0.07%	0.32% ± 0.07%
INDEL	1KG phase III	83	774 476 ± 489	755 367 ± 1196	97.28% ± 0.15%	2.72% ± 0.15%

**Figure 1:**
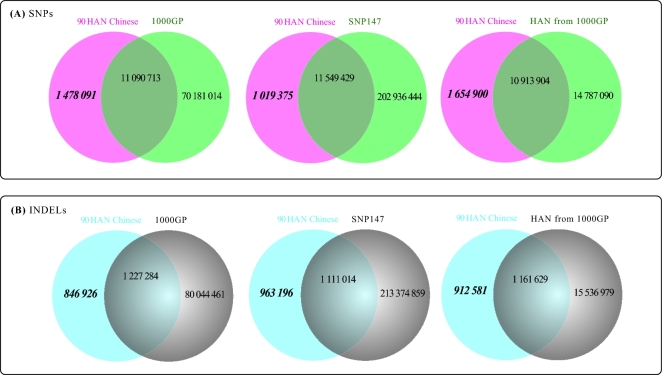
The results of novel variants. (**A**) The novel SNPs when comparing the SNP set of our 90 HAN Chinese with those of 1000GP, SNP147, or HAN Chinese from 1000GP (CHB+CHS). (**B**) The novel InDels when comparing InDels of our 90 HAN Chinese with those of 1000GP, SNP147, or HAN Chinese from 1000GP (CHB+CHS).

**Figure 2:**
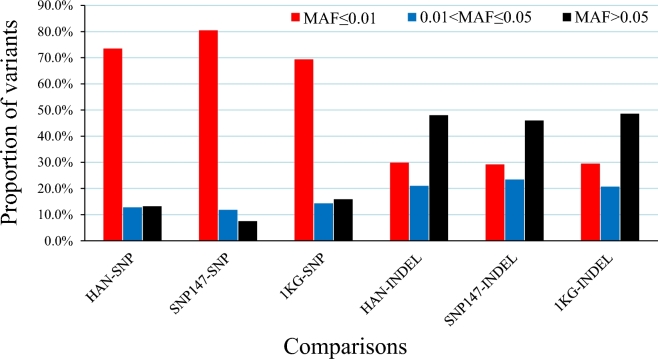
The proportion distribution of novel SNPs and InDels against minor allele frequency. HAN-SNP: the comparison of the SNP set generated from our 90 Han Chinese and the SNP set from the Han Chinese of the 1000GP. SNP147-SNP: the comparison of SNPs between our 90 Han Chinese and the dbSNP build147. 1KG-SNP: the comparison of the SNP sets between 90 Han Chinese and 1KG phase III release. HAN-INDEL: the comparison of INDELs between 90 Han Chinese and Han Chinese from 1000GP. SNP147-INDEL: the comparison of INDELs between 90 Han Chinese and dbSNP build147. 1KG-INDEL: the comparison of INDELs between 90 Han Chinese and 1000GP phase III release.

In order to highlight the strengths of deep sequencing and to mine potential novel variants particular to Han Chinese, we compared our variant set to the respective sets of the 1000GP, the Han Chinese subset of the 1000GP, and the dbSNP (build 147). The results showed that a large amount of novel variants were found in all 3 comparisons. In the comparison to the set of the Han Chinese, the 1000GP, 13.17% of the SNPs, and 43.99% of the InDels proved novel (Fig. 1). Among these novel variants, more than 85% of the SNPs and 51% of the InDels can be classified as low frequency or rare, and the proportion of rare variants was larger than that of low-frequency variants. This feature of the variants was particularly visible in the comparison between our set and Han Chinese set of 1000GP, in which the low-frequency SNPs (MAF < 5%) account for 91.7% of the novel set, and in which the rare variants are as high a fraction as 56.8% (Fig. 2).

In summary, the deep genome sequencing of 90 Han Chinese individuals presents a powerful performance in characterizing novel variants, of which the majority are low frequency or rare. It also lays a foundation to explore the potential functions of these variants in further studies.

### Genome assembly

We used SOAPdenovo2 (SOAPdenovo2, RRID:SCR_014986) [20] to assemble the genome of each individual, based on libraries with hierarchical insert size. For the genomic data of each individual, we performed several analysis steps before genome assembly. These steps are as follows: (i) filtering of low-quality reads, correcting of base calling errors; (ii) filtering out reads with adapters (match length ≥10 bp, mismatch ≤ 3); (iii) filtering out reads with fractions of *n* larger than 10%; (iv) filtering out reads with low-quality base rates in excess of 40%; (v) removal of duplicated reads produced in polymerase chain reaction amplification; (vi) calculation of k-mer frequency of all reads in order to generate frequency tables; and (vii) removal of reads with low-frequency k-mers. Detailed protocol information about *de novo* assembly has been described and saved on protocols.io [21].

We finally used reads with insert sizes of less than 2k to assemble the contigs, and we used all reads to assemble scaffolds. The k-mer size of *de novo* assembly was set as 63, and the merge level was 2. Counting the assembled genomes, the average genome size was 2 951 301 058 ± 12 168 854 bps, the average contigs N50 was 2865 ± 97 bps, and the average contig size was 49 339 ± 6088 bps.

### Structural variations calling and genotyping

**Figure 3:**
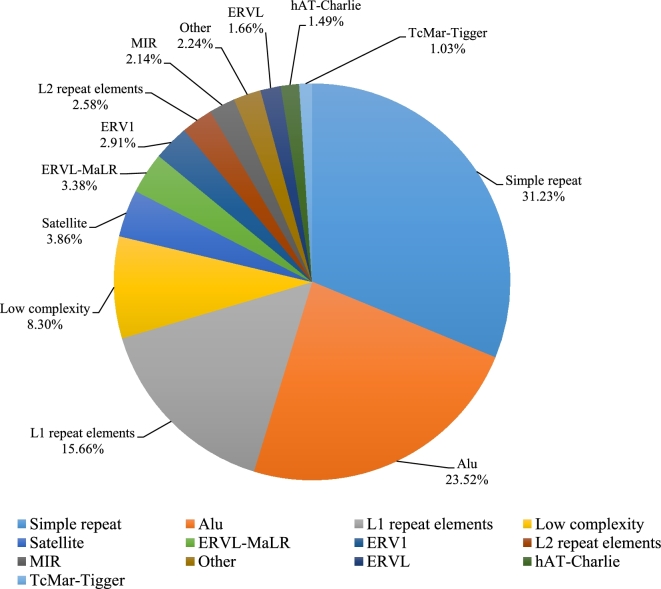
Annotation results of deletion breakpoints. Combine-repeat: combining repeat types with low frequency; L1: L1 repeat elements; L2: L2 repeat elements; MIR: mammalian interspersed repetitive (MIR) element; hAT-Charlie: one kind of DNA transposons.

We here applied an integrative strategy to identify the SVs of Han Chinese people, including multiple current algorithms. The assembly-based method (SOAPsv) [22] was firstly employed in SV calling. We finally obtained a total of 26 142 SVs, containing 12 772 insertions and 13 370 deletions. The average number for each individual is 3102 ± 190. Besides this, several other methods were then applied to call SVs, including Pindel [23], CNVnator [24], Breakdancer [25], and Genome STRiP [26]. More detailed protocol information on SV calling can be found on the protocols.io platform [27]. We then merged all of the deletions in several SV sets according to their breakpoints to obtain an integrative deletion set. These deletions were genotyped in the tool Genome STRiP. We then discarded the SVs with large proportions of un-qualified genotypes (>10%), which included genotypes of low quality (phred-scaled likelihood score lower than 13) and those that were missing. After the above steps, a total of 24 369 deletions passed this filtering. After annotation by RepeatMasker (RepeatMasker, RRID:SCR_012954) [28], more than 70% of these deletions were found to be in the simple repeat (31.23%), the Alu (23.52%), and the L1 repeat elements (15.66%) (Fig. 3).

**Figure 4:**
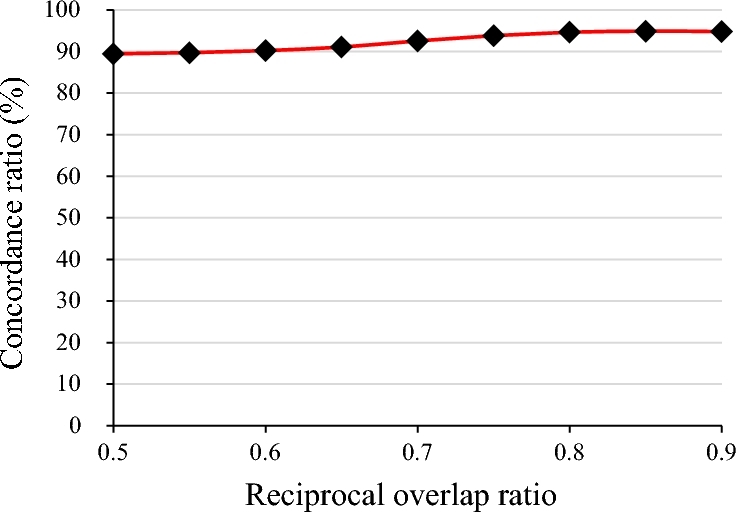
The concordance rates of SVs between the 1000 Genomes Project and 90 Han Chinese.

Length distribution showed an enrichment of short deletions (<5 Kb), accounting for 76.77% of all deletions. Only 0.45% of these proved larger than 500 Kb. Based on the criterion of 50% reciprocal overlap [12], we found that 61.58% of our deletion set was novel with respect to the SVs of the 1000GP; 20% of the novel deletions were low frequency (MAF < 5%). We then evaluated the genotype concordance by comparing with the SV set of the 1000GP. We compared genotypes of the overlapping SVs in both sets. Concordance rates reached 89.45% ± 0.004%. It was as high as 94.90% when the proportion of reciprocal overlap was set at 85% (Fig. 4).

We built a more comprehensive SV catalogue for Han Chinese people using the above integrative strategy, including assembly-based method, Pindel, CNVnator, Breakdancer, and Genome STRiP. This catalogue harbors significantly more SVs than that of the Han Chinese from 1000GP (∼7700), and the majority of them are novel (17 927, 73.56%). The set overlap in SVs between the 2 sets evidences high concordance of genotype. This provides a valuable data panel for use in research about human diversity and genetic diseases.

### Availability and requirements

Project name: WGS of Han Chinese genomes

Project home page: https://github.com/HaoxiangLin/WGS_of_Han_Chinese_genomes

Operating system(s): Linux

Programming language: Shell, Perl, Java, and C++

Other requirements: BWA, 0.6.1; SOAPsv, 1.02; SOAPdenovo2; PINDEL, 0.2.4t; cnvnator, 0.2.7; Breakdancer-max, 1.2; Genome STRIP, v. 1.0; SAMtools, 0.1.18; Picard, 1.61; GATK, 2.7; BEAGLE, v. 3

License: GNU General Public License v.3.0 (GPLv3)

## Availability of supporting data

The raw fastq format data were deposited at EBI with the project accession number PRJEB11005, and the secondary accession number ERP012319. VCFs have been archived at EMBL-EBI under accession number PRJEB20820. Datasets further supporting the results of this data note are available in the *GigaScience* database, *Giga*DB [29]. Protocols are also available from protocols.io [18, 21, 27].

## Abbreviations

1000GP: 1000 Genomes Project; CHB: Han Chinese in Beijing; CHS: Southern Han Chinese; FDR: false discovery rate; GATK: Genome Analysis Tool Kit; GWAS: genome-wide association study; HRC: Haplotype Reference Consortium; MAF: minor allele frequency; SV: structural variant.

## Competing interests

The authors declare that they have no competing interests.

## Author contributions

X.G., X.X., H.Y., J.W., J.W., and X.L. conceived this project. X.G. and H.L. collected the samples, isolated the genomic DNA, and constructed the DNA libraries. T.L., H.L., X.G., W.Z., and M.Y. performed the genome analysis. L.C.A.M.T. provided advice. H.L., T.L., and X.G. helped deposit and curate the datasets in *Giga*DB. T.L., X.G., and L.C.A.M.T. wrote the article. All authors discussed the project and data. All authors read and approved the final manuscript.

## Supplementary Material

GIGA-D-16-00115_Original-Submission.pdf

GIGA-D-16-00115_Revision-1.pdf

GIGA-D-16-00115_Revision-2.pdf

GIGA-D-16-00115_Revision-3.pdf

GIGA-D-16-00115_Revision-4.pdf

Response-to-Reviewer-Comments_Original-Submission.pdf

Response-to-Reviewer-Comments_Revision-1.pdf

Response-to-Reviewer-Comments_Revision-2.pdf

Response-to-Reviewer-Comments_Revision-3.pdf

Reviewer-1-Report-(Original-Submission).pdf

Reviewer-1-Report-(Revision-1).pdf

Reviewer-2-Report-(Original-Submission).pdf

Reviewer-2-Report-(Revision-1).pdf

Reviewer-3-Report-(Original-Submission).pdf

Reviewer-4-Report-(Original-Submission).pdf

Reviewer-4-Report-(Revision-1).pdf

## References

[1] Durbin RM, Altshuler DL, Durbin RM A map of human genome variation from population-scale sequencing. Nature 2010;467(7319):1061–73. 20981092 10.1038/nature09534 PMC3042601

[2] Mcvean GA, Altshuler DM, Durbin RM An integrated map of genetic variation from 1,092 human genomes. Nature 2012;491(7422):56–65. 23128226 10.1038/nature11632 PMC3498066

[3] Auton A, Abecasis GR, Altshuler DM A global reference for human genetic variation. Nature 2015;526(7571):68–74. 26432245 10.1038/nature15393 PMC4750478

[4] Mccarthy S, Das S, Kretzschmar W A reference panel of 64,976 haplotypes for genotype imputation. Nat Genet 2016;48(10):1279–83. 27548312 10.1038/ng.3643 PMC5388176

[5] Li Y, Sidore C, Kang HM Low-coverage sequencing: implications for design of complex trait association studies. Genome Res 2011;21(6):940–51. 21460063 10.1101/gr.117259.110 PMC3106327

[6] Walter K, Min JL, Huang J The UK10K project identifies rare variants in health and disease. Nature 2015;526(7571):82–90. 26367797 10.1038/nature14962 PMC4773891

[7] Bizon C, Spiegel M, Chasse SA Variant calling in low-coverage whole genome sequencing of a Native American population sample. BMC Genomics 2014;15 1:85. 24479562 10.1186/1471-2164-15-85 PMC3914019

[8] Yi X, Liang Y, Huerta-Sanchez E Sequencing of 50 human exomes reveals adaptation to high altitude. Science 2010;329(5987):75–78. 20595611 10.1126/science.1190371 PMC3711608

[9] Auer PL, Lettre G Rare variant association studies: considerations, challenges and opportunities. Genome Med 2015;7(1):16. 25709717 10.1186/s13073-015-0138-2 PMC4337325

[10] Feuk L, Carson AR, Scherer SW Structural variation in the human genome. Nat Rev Genet 2006;7(2):85–97. 16418744 10.1038/nrg1767

[11] Pang AW, Macdonald JR, Pinto D Towards a comprehensive structural variation map of an individual human genome. Genome Biol 2010;11(5):R52. 20482838 10.1186/gb-2010-11-5-r52 PMC2898065

[12] Sudmant PH, Rausch T, Gardner EJ An integrated map of structural variation in 2,504 human genomes. Nature 2015;526(7571):75–81. 26432246 10.1038/nature15394 PMC4617611

[13] English AC, Salerno WJ, Hampton OA Assessing structural variation in a personal genome—towards a human reference diploid genome. BMC Genomics 2015;16(1):286. 25886820 10.1186/s12864-015-1479-3 PMC4490614

[14] Li Y, Zheng H, Luo R Structural variation in two human genomes mapped at single-nucleotide resolution by whole genome de novo assembly. Nat Biotechnol 2011;29(8):723–30. 21785424 10.1038/nbt.1904

[15] Li H, Durbin R Fast and accurate long-read alignment with Burrows–Wheeler transform. Bioinformatics 2010;26(5):589–95. 20080505 10.1093/bioinformatics/btp698 PMC2828108

[16] Mckenna A, Hanna M, Banks E The Genome Analysis Toolkit: a MapReduce framework for analyzing next-generation DNA sequencing data. Genome Res 2010;20(9):1297–303. 20644199 10.1101/gr.107524.110 PMC2928508

[17] Depristo MA, Banks E, Poplin R A framework for variation discovery and genotyping using next-generation DNA sequencing data. Nat Genet 2011;43(5):491–8. 21478889 10.1038/ng.806 PMC3083463

[18] Lin H SNP INDEL calling. protocols.io 2017. http://dx.doi.org/10.17504/protocols.io.grkbv4w.

[19] Wang K, Li M, Hakonarson H ANNOVAR: functional annotation of genetic variants from high-throughput sequencing data. Nucleic Acids Res 2010;38(16):e164. 20601685 10.1093/nar/gkq603 PMC2938201

[20] Luo R, Liu B, Xie Y SOAPdenovo2: an empirically improved memory-efficient short-read de novo assembler. Gigascience 2012;1(1):18. 23587118 10.1186/2047-217X-1-18 PMC3626529

[21] Lin H Soapdenovo Genome assembly. protocols.io 2017 http://dx.doi.org/10.17504/protocols.io.gr3bv8n.

[22] Li Y, Zheng H, Luo R Structural variation in two human genomes mapped at single-nucleotide resolution by whole genome de novo assembly. Nat Biotechnol 2011;29(8):723–30. 21785424 10.1038/nbt.1904

[23] Ye K, Schulz MH, Long Q Pindel: a pattern growth approach to detect break points of large deletions and medium sized insertions from paired-end short reads. Bioinformatics 2009;25(21):2865–71. 19561018 10.1093/bioinformatics/btp394 PMC2781750

[24] Abyzov A, Urban AE, Snyder M CNVnator: an approach to discover, genotype, and characterize typical and atypical CNVs from family and population genome sequencing. Genome Res 2011;21(6):974–84. 21324876 10.1101/gr.114876.110 PMC3106330

[25] Chen K, Wallis JW, Mclellan MD BreakDancer: an algorithm for high-resolution mapping of genomic structural variation. Nat Methods 2009;6(9):677–81. 19668202 10.1038/nmeth.1363 PMC3661775

[26] Handsaker RE, Korn JM, Nemesh J Discovery and genotyping of genome structural polymorphism by sequencing on a population scale. Nat Genet 2011;43(3):269–76. 21317889 10.1038/ng.768 PMC5094049

[27] Lin H Structure variation detection. protocols.io 2017. http://dx.doi.org/10.17504/protocols.io.gr4bv8w.

[28] Saha S, Bridges S, Magbanua ZV Computational approaches and tools used in identification of dispersed repetitive DNA sequences. Tropical Plant Biol 2008;1(1):85–96.

[29] Lan T-M, Lin H-X, Zhu W-J Supporting data for “Deep whole-genome sequencing of 90 Han Chinese genomes.” GigaScience Database 2017 http://dx.doi.org/10.5524/100302. 10.1093/gigascience/gix067 PMC5603764 28938720

