# Correction to: Deep whole-genome sequencing of 90 Han Chinese Genomes

**DOI:** 10.1093/gigascience/giz001

**Published:** 2019-02-25

**Authors:** Tianming Lan, Haoxiang Lin, Wenjuan Zhu, Laurent Christian Asker Melchior Tellier, Mengcheng Yang, Xin Liu, Jun Wang, Jian Wang, Huanming Yang, Xun Xu, Xiaosen Guo

**Affiliations:** 1 BGI-Shenzhen, Building 11, Beishan Industrial Zone, Yantian District, Shenzhen, 518083, China; 2 BGI Genomics, BGI-Shenzhen, Building NO. 7, BGI Park, No. 21 Hongan 3rd Street, Yantian District, Shenzhen, 518083, China; 3 Department of Biology, University of Copenhagen, Nørregade 10, PO Box 2177 1017 Copenhagen, Denmark; 4 James D. Watson Institute of Genome Sciences, 866 Yuhangtang Road, Hangzhou, Zhejiang Province, 310058, P. R. China; 5 Shenzhen Key Laboratory of Neurogenomics, BGI-Shenzhen, Building 11, Beishan Industrial Zone, Yantian District, Shenzhen, 518083, China

This is a correction to:

GigaScience, Volume 6, Issue 9, 1 September 2017, gix067,

https://doi.org/10.1093/gigascience/gix067

In the original version of the article ‘Deep whole-genome sequencing of 90 Han Chinese Genomes,’ by Tianming Lan et al. [1], the names of the 4th author (Tellier Christian Asker Melchior Laurent) was in the incorrect order. In the address, ‘Building 11’ of the BGI campus in Shenzhen was also incorrectly referred to as ‘Build 11.’ The 4th author name and the address have been corrected, and the authors apologize for the error.
