# Supplementary material for: Deep whole-genome sequencing of 90 Han Chinese genomes
Source: Gigascience. 2017 Jul 31;6(9):1–7. doi: 10.1093/gigascience/gix067 (PMC5603764; doi:10.1093/gigascience/gix067)
Supplement: GIGA-D-16-00115_Original-Submission.pdf [file gix067_GIGA-D-16-00115_Original-Submission.pdf]

Manuscript
Click here to download Manuscript Deep Whole-genome Sequencing of 90 Han Chinese - v1.1_Laur.docx
Click here to view linked References

Deep whole-genome sequencing of 90 Han Chinese genomes

Tianming Lan[1†], Haoxiang Lin[1†], Wenjuan Zhu[1], Laurent Christian Asker Melchior Tellier[1,2], Xin Liu[1], Jun Wang[1,2], Jian Wang[1,4], Huanming Yang[1,4], Xun Xu[1], Xiaosen Guo[1,2,3*]

*Correspondence: guoxs@genomics.cn

†Equal contributors

[1]BGI-Shenzhen, Shenzhen 518083, China

[2]Department of Biology, University of Copenhagen, Ole Maaløes Vej 5, 2200 Copenhagen, Denmark.

[3]Shenzhen Key Laboratory of Neurogenomics, BGI-Shenzhen, Shenzhen 518083, China.

Abstract

Background: Next generation sequencing provides a full view of human genetic information. However, the focus of previous studies has been primarily on low coverage data, due to the high cost of sequencing. Imputation technology provides an opportunity to obtain the missing genotypes, and enables sequencing strategies of large sample size at low sequencing depth. Imputation power is mainly determined by the quality of the reference panel used, and 1000 Genomes Project data is not always sufficient for this purpose, due to low sequencing depth, especially for Chinese populations. There is a strong need for the generation of sets of deep coverage sequencing data on the Chinese population, in order to construct a high quality reference panel.

Findings: We have performed very deep, whole genome sequencing (~80X) on 90 unrelated individuals of Chinese ancestry. 38 samples were gathered from Hunan province, 7 from Fujian province, 45 samples from Beijing. We built the first variant catalog of the Han Chinese by deep sequencing. We identified 12,568,804 SNPs, 2,074,734 INDELs and 108,434 Structural variations by de novo assembly and re-sequencing methods.

Conclusions: Compared to the low pass sequencing strategy used in the 1000 Genomes Project, this high depth sequencing of Han Chinese will provide a more accurate and more complete reference panel, which can improve imputation accuracy, especially for Chinese and other East Asian human populations. It will be a valuable resource for promoting Chinese genetics research.

Key Words: High-coverage Whole-genome Sequencing, Han Chinese genomes, *Denovo* assembly, Genetic variations

Data Description

Backgrounds

NGS has been widely used in human genetics research. The 1000 Genomes Project has elucidated patterns and distributions of variation in human genomes all over the world, supplying researchers with a global set of genetic reference panels [1-3]. This has benefited human disease research, such as Genome Wide Association Studies (GWAS). GWAS has been powerful in uncovering associations between phenotype and genotype, especially in cases of human disease [4-6]. Generally, GWAS needs thousands or tens thousands of samples to achieve sufficient power to obtain confident association results [7]. But deep sequencing on large sample sizes is prohibitively costly, for most applications.

However, imputation technology enables sequencing strategies which aim for large sample sizes, but low and inexpensive sequencing depth. This method mainly relies upon dense genotyping data, called a human reference panel, and in particular linkage disequilibrium (LD) relationships between variation upon known genotypes, to impute genotypes for which direct variant calling information is missing [8]. Imputation power of a SNP of interest is mainly determined by the quality of the reference panel, the LD of the SNP of interest to neighboring SNPs, the density of the SNPs surrounding the SNP of interest, and the genotyping accuracy of SNPs in the reference panel from which the imputation regimen draws inference. However, each sample from the 1000 Genomes Project has an average sequencing depth of only 5~7X, which is low that heterozygous variants are frequently incorrectly called, and false variants are commonly introduced. Also, the 1000 Genomes data is not suitable imputation panel for specifically Chinese populations, due to regional genetic characteristics of the samples gathered for the panel.

In our study, we have sequenced 90 Chinese samples at an average sequencing depth of ~80X. 45 samples were gathered from southern China, and 45 samples from northern China. These samples encompass a more representative extract of Han Chinese genetic variation. High-depth sequencing of these samples has supplied us with highly confident variant calls upon the samples, resulting in a very high quality reference panel. This data can later be used as a valuable resource for performing imputation upon lower pass sequencing samples, inferring high quality imputation results for the appropriate population.

Samples

Genomic DNA of 90 unrelated Chinese individuals were collected from Coriell Institute cell lines. 38 samples were gathered from Hunan province, 7 from Fujian province, 45 from Beijing. Finally, 45 samples were from southern China and the other 45 samples were from northern China. All 90 samples were also characterized as part of the 1000 Genomes Project.

Ethics Statement

Participants in our study were all from 1000 Genome Project, and all individuals consented that their genomic data can be used in the analysis of the project and can be freely distributed for future studies. And the public distribution of the sequencing data and genetic variations and genotypes was also been explicitly consented. The study had been also approved by the Institutional Review Board on Bioethics and Biosafety.

Sequencing

Library preparation was done by following the manufacturer's instructions (Illumina). We performed the cluster generation using the Illumina cluster station, and the workflow was as follows: template hybridization, isothermal amplification, linearization, blocking, denaturation and sequencing primer hybridization. The fluorescent images were processed to sequences, using the standard Illumina base-calling pipeline. We build 5 ranks of lanes with different insert size length (170bp, 500bp, 2kb, 5kb, 10kb, 20kb) (Table1). The average sequencing depth of CHS was 71.87 ± 23.52, and that of CHB was 82.36 ± 14.13. The average genome coverage of CHS was 99.65% ± 0.34%, and that of CHB was 99.60% ± 0.30% (Table 2).

SNP/INDEL discovery

The read alignments were performed using the *aln* algorithm of BWA 0.6.2-r126[9], and we used reads with insert size ranging from 180bp to 500bp, aligning against human genome reference version Hg19. We used GATK (version 2.8.1)[10] to remove duplications, realign around indels, and to recalibrate alignment quality scores to our machines. SNP and INDEL calls were performed using GATK UnifiedGenotyper. A total of 15,240,533 raw SNPs were called. After VQSR filtering, we arrived at 12,568,804 filtered SNPs, and 2,074,734 filtered INDELs. Of these variants, 12,536,571 SNPs and 1,473,062 INDELs were bi-allelic, 4,535,591 were rare variants, 2,875,420 were low frequency variants, and 6,598,622 were common variants.

Genotype calling

We used the method of genotype likelihood estimation to call genotypes. We combined all SNP sites of each individual into a list. We extracted the genotyping likelihoods of each variant site, in each individual, using SAMtools (0.1.8, r613)[11]. We estimated Maximum Likelihood (ML) of each allele using the expectation–maximization method. Next, we filtered the SNPs by MAF < 0.005, and LRT < 20, to remove low quality genotypes. We performed Beagle imputation, using the genotype likelihood as input. We also carried out several tests to filter unusual genotypes, such as ones with base quality strand bias,

low depth, poor mappability, homopolymers, allele balances, Hardy-Weinberg equilibrium. For more details, please refer to the Table 3.

Genome assembly

We used the SOAPdenovo2 algorithm[12] to assembly each individual genome *denovo*. Before genome assembly, for the data of each individual, we undertook several processes to filter low quality reads and correct base calling errors. We filtered reads with adapters (match length >= 10bp, mismatch <=3), filtered reads with the percent of N larger than 10%, filtered reads with more than 40% low quality bases, deduplicated reads to remove probable PCR duplications, calculated k-mer frequency of all reads to generate frequency tables and remove reads with low frequency k-mers.

We finally used reads with insert size below 2k to assemble the contigs, and used all reads to assemble the scaffolds. The k-mer size of *denovo* assembly was 63, and the merge level was 2. In total, 90 genome assembly results were generated. The average genome size was 2,951,301,058 ± 12,168,854 bps. The average N50 was 2,865 ± 97 bps. The average contig size was 49,339 ± 6,088 bps.

Structural variations calling and genotyping

We aligned the contigs to the hg19 reference genome to discover Structural Variants (SV). Contigs of more reliable and with less assembly errors can make the SV calling more reliable. Two stages were involved in SV calling: the first phase is fast mapping, which using the BWA-SW to locate contigs in the reference genome. The second phase is the LASTZ aligner, to hold large gaps on the alignments. We discover SVs by large gap discovery in sequence alignments. We genotype SVs by combining read depth and coverage in the SV regions. An average number of 3102 ± 190 SVs were detected for each sample. In total, 108,434 candidate SVs were finally called. We used the software Genome STRiP [13] to genotype all these SVs in the Chinese population, and 96,637 SVs were finally successfully genotyped. Most of the deletion breakpoints were found to be distributed in the simple repeat and the Alu regions (Figure 1). 36,831 SVs were located in the gene region, 35,723 SVs were in the intron, 7,134 in the CDS regions, 5,100 in 5-UTR regions and 4,686 were located in the 3-UTR regions.

Notes

Tianming Lan and Haoxiang Lin contributed equally to this work.

Abbreviations

NGS, Next Generation Sequencing; GWAS, Genome-Wide Association Study; CHB, Han Chinese in Beijing; CHS, Southern Han Chinese; MAF, Minor Allele Frequency; LRT, Likelihood Ratio Test;

GATK, Genome Analysis Tool Kit; VQSR, Variant Quality Score Recalibration.

Acknowledgements

We thank for the support of Shenzhen Municipal of Government of China (CXB201108250094A).

Availability of supporting data

The raw fastq format data were deposited at EBI with the project accession PRJEB11005, and the secondary accession ERP012319. This datasets in this article are available in the GigaDB database.

Authors' contributions

XX, HY, JW, JW and XL conceived this project. HL and XG collected the samples, isolated the genomic DNA and constructed the DNA libraries. XG, TL, and WZ performed the genome analysis. LCAMT provided advice. XG and TL submitted the dataset to GigaDB. TL, XG and LCAMT wrote the article. All authors discuss the project and data. All authors read and approved the final manuscript.

Competing interests

The authors declare that they have no competing interests.

Author details

[1]BGI-Shenzhen, Shenzhen 518083, China. [2]Department of Biology, University of Copenhagen, Ole Maaløes Vej 5, 2200 Copenhagen, Denmark. [3]Shenzhen Key Laboratory of Neurogenomics, BGI-Shenzhen, Shenzhen 518083, China. [4]James D. Watson Institute of Genome Sciences, Hangzhou 310058, China

Reference

1. Abecasis GR, Altshuler D, Auton A, Brooks LD, Durbin RM, Gibbs RA, Hurles ME, McVean GA: **A map of human genome variation from population-scale sequencing**. *Nature* 2010, **467**(7319):1061-1073.

2. Abecasis GR, Auton A, Brooks LD, DePristo MA, Durbin RM, Handsaker RE, Kang HM, Marth GT, McVean GA: **An integrated map of genetic variation from 1,092 human genomes**. *Nature* 2012, **491**(7422):56-65.

3. Auton A, Brooks LD, Durbin RM, Garrison EP, Kang HM, Korbel JO, Marchini JL, McCarthy S, McVean GA, Abecasis GR: **A global reference for human genetic variation**. *Nature* 2015, **526**(7571):68-74.

4. Todd JA, Walker NM, Cooper JD, Smyth DJ, Downes K, Plagnol V, Bailey R, Nejentsev S, Field SF, Payne F *et al*: **Robust associations of four new chromosome regions from genome-wide analyses of type 1 diabetes**. *Nat Genet* 2007, **39**(7):857-864.

5. Parkes M, Barrett JC, Prescott NJ, Tremelling M, Anderson CA, Fisher SA, Roberts RG, Nimmo ER, Cummings FR, Soars D *et al*: **Sequence variants in the autophagy gene IRGM and multiple other replicating loci contribute to Crohn's disease susceptibility**. *Nat Genet* 2007, **39**(7):830-832.

6. Duerr RH, Taylor KD, Brant SR, Rioux JD, Silverberg MS, Daly MJ, Steinhart AH, Abraham C, Regueiro M, Griffiths A *et al*: **A genome-wide association study identifies IL23R as an inflammatory bowel disease gene**. *Science* 2006, **314**(5804):1461-1463.

7. Pearson TA, Manolio TA: **How to interpret a genome-wide association study**. *Jama* 2008, **299**(11):1335-1344.

8. Browning BL, Browning SR: **Genotype Imputation with Millions of Reference Samples**. *Am J Hum Genet* 2016, **98**(1):116-126.

9. Li H, Durbin R: **Fast and accurate long-read alignment with Burrows-Wheeler transform**. *Bioinformatics* 2010, **26**(5):589-595.

10. DePristo MA, Banks E, Poplin R, Garimella KV, Maguire JR, Hartl C, Philippakis AA, del Angel G, Rivas MA, Hanna M *et al*: **A framework for variation discovery and genotyping using next-generation DNA sequencing data**. *Nat Genet* 2011, **43**(5):491-498.

11. Li H, Handsaker B, Wysoker A, Fennell T, Ruan J, Homer N, Marth G, Abecasis G, Durbin R, Genome Project Data Processing S: **The Sequence Alignment/Map format and SAMtools**. *Bioinformatics* 2009, **25**(16):2078-2079.

12. Luo R, Liu B, Xie Y, Li Z, Huang W, Yuan J, He G, Chen Y, Pan Q, Liu Y *et al*: **SOAPdenovo2: an empirically improved memory-efficient short-read de novo assembler**. *GigaScience* 2012, **1**(1):18.

13. Handsaker RE, Korn JM, Nemesh J, McCarroll SA: **Discovery and genotyping of genome structural polymorphism by sequencing on a population scale**. *Nat Genet* 2011, **43**(3):269-276.

Table
Click here to download Table Table 1 The sequencing depth of different library insert size.docx

Table 1 The sequencing depth of different library insert size

**Library insert size**	**Sequencing depth (fold)**	**Standard deviation**
180bp	51.78	8.11
500bp	12.74	2.54
2,000bp	5.01	1.08
5,000bp	5.02	2.08
10,000bp	5.62	2.22
20,000bp	6.68	2.52
<1,000bp	64.52	8.11
>1,000bp	22.33	3.90
Total	86.85	8.53

*Sequencing depth is calculated as total sequencing base / 3e10.

Table

Click here to download Table Table2 Deep whole genome sequencing data of 90 Chinese samples.docx

Table2 Deep whole genome sequencing data of 90 Chinese samples

	CHS	CHB	Total
Number of individuals	45	45	90
Raw bases (Gb)	231.61±72.61	264.24±44.92	247.69±56.54
Mapped bases (Gb)	212.35±68.96	243.28±41.81	227.57±53.74
Average sequencing depth (fold)	71.87±23.52	82.36±14.13	77.02±18.37
Fraction of target covered >= 1X (%)	99.65±0.34	99.60±0.30	99.62±0.33
Fraction of target covered >= 4X (%)	99.40±0.44	99.37±0.40	99.39±0.42
Fraction of target covered >= 8X (%)	99.05±0.51	99.08±0.49	99.07±0.53
Fraction of target covered >= 16X (%)	98.24±0.53	98.35±0.61	98.29±0.60
Fraction of target covered >= 32X (%)	95.69±2.15	96.48±1.72	96.06±2.53

Click here to download Table Table 3 Criteria of filtering for genotypes.docx

Table 3 Criteria of filtering for genotypes

**Filter**	**Criteria**	**Removed**	**In dbSNP (v135)**
Mappability	Mappability score[a] < 0.5	390,289	170,804
Strand bias	Fisher exact test *P*-value < 1.0e-10 & log2 odds ratio > 4 or < -4	622,960	80,902
Depth	Average depth < 4 or > 200	165,301	43,442
Allelic banlance	Binomial test *P*-value < 1.0e-10 and bias[b] > 0.5	778,228	240,230
Homopolymer	Homopolymer run[c] > 6	755,422	343,549
Basequality	Wilcox ranksum test P-value < 1.0e-7 & difference[d] > 0.1	452,096	50,546
All above filters	Any one	2,179,474	613,851

a: mappability score measures the accessibility of a genomic locus by short read sequencing. b: the absolute difference of reads number between major allele and minor allele. c: the length of the homopolymer of a SNP site. d: the absolute difference of base quality scores between major allele and minor allele.

Figure

Click here to download Figure Figure1 The annotation results of deletion breakpoints.docx

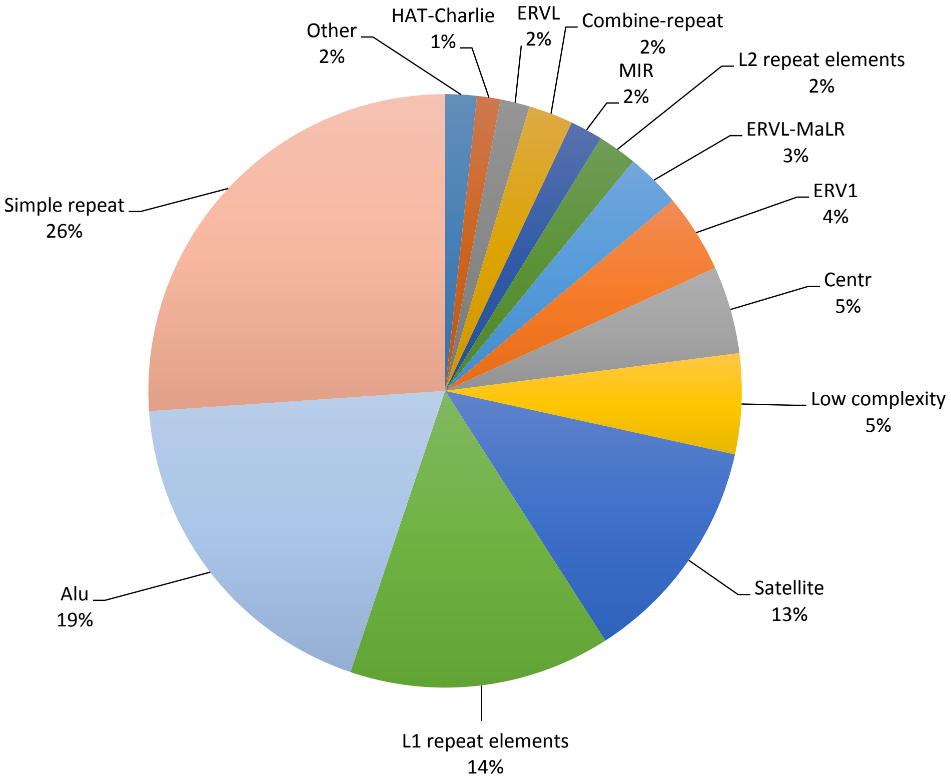

Figure 1 The annotation results of deletion breakpoints. Combine-repeat: combining repeat types with low frequency; L1: L1 repeat elements; L2: L2 repeat elements; MIR: mammalian interspersed repetitive (MIR) element; hAT-Charlie: one kind of DNA transposons.
