# Supplementary material for: Deep whole-genome sequencing of 90 Han Chinese genomes
Source: Gigascience. 2017 Jul 31;6(9):1–7. doi: 10.1093/gigascience/gix067 (PMC5603764; doi:10.1093/gigascience/gix067)
Supplement: GIGA-D-16-00115_Revision-3.pdf [file gix067_GIGA-D-16-00115_Revision-3.pdf]

Manuscript

Click here to download Manuscript Deep Whole-genome Sequencing of 90 Han Chinese.docx

Deep whole-genome sequencing of 90 Han Chinese genomes

Tianming Lan[1†], Haoxiang Lin[2†], Wenjuan Zhu[2], Laurent Christian Asker Melchior Tellier[1,3], Mengcheng Yang[1], Xin Liu[1], Jun Wang[1,3], Jian Wang[1,4], Huanming Yang[1,4], Xun Xu[1], Xiaosen Guo[1,3,5*]

*Correspondence: guoxs@genomics.cn

†Equal contributors

[1]BGI-Shenzhen, Shenzhen 518083, China.

[2]BGI Genomics, BGI-Shenzhen, Shenzhen 518083, China.

[3]Department of Biology, University of Copenhagen, Ole Maaløes Vej 5, 2200 Copenhagen, Denmark.

[4]James D. Watson Institute of Genome Sciences, Hangzhou 310058, China.

[5]Shenzhen Key Laboratory of Neurogenomics, BGI-Shenzhen, Shenzhen 518083, China.

Abstract

Background: Next generation sequencing provides a high-resolution insight into human genetic information. However, the focus of previous studies has primarily been on low-coverage data, due to the high cost of sequencing. Although the 1000 Genomes Project and the Haplotype Reference Consortium have both provided powerful reference panels for imputation, low frequency and novel variants remain difficult to discover and call with accuracy on the basis of low-coverage data. Deep sequencing provides an optimal solution for the problem of these low frequency and novel variants. Although whole exome sequencing is also a viable choice for exome regions, it cannot account for noncoding regions, sometimes resulting in the absence of important, causal variants. For Han Chinese populations, the majority of variants have been discovered based upon low-coverage data from the 1000 Genomes Project. However, high-coverage, whole genome sequencing data is limited for any population, and a large amount of low-frequency, population-specific variants remains uncharacterized.

Findings: We have performed whole genome sequencing at high depth (~80X) of 90 unrelated individuals of Chinese ancestry, collected from the 1000 Genome Project samples, including 45 North Han Chinese and 45 South Han Chinese samples. 83 of these 90 have been sequenced by the 1000 Genomes Project. We have identified 12,568,804 single nucleotide polymorphisms, 2,074,210 short InDels and 26,142 structural variations from these 90 samples. Compared to the Han Chinese data from the 1000 Genomes Project, we have found 7,000,629 novel variants with low frequency (defined as minor allele frequency < 5%), including 5,813,503 SNPs, 1,169,199 InDels, and 17,927 structural variants.

Conclusions: Using deep sequencing data, we have built a greatly expanded spectrum of genetic variation for the Han Chinese genome. Compared to the 1000 Genomes Project, this Han Chinese deep sequencing data enhances characterization of a large number of low-frequency, novel variants. This will be a valuable resource for promoting Chinese genetics research and medical development. Additionally, it will provide a valuable supplement for the 1000 Genomes Project, as well as for other human genome projects.

Key Words: High-coverage Whole-genome Sequencing, Han Chinese genomes, *Denovo* assembly, Genetic variations

Data Description

Background

Next Generation Sequencing (NGS) has been increasingly and widely utilized in human genomics research compared to what has been previously possible, and in particular for Genome-Wide Association Studies (GWAS). The 1000 Genomes Project (1000GP) had distributed a standard pattern of over 88 million variants, providing for research use a global genetic reference panel [1-3]. The Haplotype Reference Consortium (HRC) has also constructed a distinct human reference panel, consisting of 39,235,157 SNPs [4]. However, most of the samples contributing to either the 1000GP or to the HRC have an average sequencing depth of only 4~8X, which makes difficult the characterization of low-frequency variants (minor allele frequency (MAF) < 5%), and especially rare variants (MAF < 1%)[5]. These two projects therefore cannot supply a high-resolution spectrum of variations for many human populations, in particular not for Han Chinese [6].

Low coverage sequencing can be used to generate a high quality variation set supplemented by imputation, though imputation will perform poorly in correctly calling rare variants, using the current set of typed markers and reference panels[6, 7]. Whole exome sequencing (WES) is a viable method to elucidate associations between rare variants and human diseases, when these are to be found in coding regions; however, WES cannot characterize non-coding regions, which encompass 98% of the human genome, and are increasingly recognized to play an important role in some human traits [8, 9]. The 1000GP, HRC and other human genome projects have generated extensive human variation catalogues, which can be used to design high-density genotyping arrays. However, these chips can be expected to miss rare or low-frequency alleles[9]. Also, for Han Chinese genomes, a large number of population-specific variants can be expected to be absent from the imputation variant panel set of 1000GP. Therefore, it is necessary to characterize the Han genome, using high-depth whole-genome sequencing.

Besides SNPs and InDels, structural variants (SVs) have also been found in recent studies [10, 11] to be associated with human diseases which contribute to human genetic diversity. 68,818 SVs have already been detected using the data of the 1000GP, and an integrated SV map for global human population has been generated [12]. However, SV identification using short reads from low coverage sequencing data remains challenging [13]. Assembly-based SV calling based on high coverage sequencing data provides a feasible and powerful method [14].

In this study, we have sequenced the genomes of 90 Han Chinese samples extracted from 1000GP, at an average sequencing depth of ~80X, including seven hitherto un-sequenced samples from 1000GP. We have built a high-resolution spectrum of genetic variation for the Han Chinese population, based on high-coverage genomic data, including SNPs, InDels and SVs. This data provides a valuable resource for performing further genomics/genetics studies in Han Chinese populations, especially for exploring the functional roles of these low-frequency novel variants. Additionally, as a valuable supplement, the data will enrich genetic variation catalogues of global human populations.

Samples

Genomic DNA was extracted from the cell lines of 90 unrelated Chinese samples from the 1000GP, currently deposited at Coriell Institute for Medical Research. 45 of these 90 samples are taken from Southern Han Chinese (CHS) and the remnant 45 are taken from Han Chinese from Beijing (CHB). Of these samples, seven were hitherto unsequenced by the1000GP, including five CHB and two CHS.

Ethics Statement

All Samples used in this study were obtained from the 1000 Genomes Project cell line collection at the Coriell Cell line repository, and were qualified by the ethics protocols of the 1000GP. According to the statement of informed consent, all samples held by the 1000GP, including these 90 Chinese individuals, have been released and shared to the public. All individuals have consented to allowing their genomic data to be used in the analyses of the project, and may be freely distributed for future research. Public distribution of the sequencing data and genotypes has also been explicitly consented to. This study had been also approved by the Institutional Review Board on Bioethics and Biosafety.

Sequencing

Library preparation was done in accordance with the manufacturer's instructions (Illumina). We performed cluster generation using the Illumina cluster station, with workflow as follows: template hybridization, isothermal amplification, linearization, blocking, denaturation and sequencing primer hybridization. Fluorescent images were processed to sequences, using the standard Illumina base-

calling pipeline. We built 5 ranks of DNA libraries with different insert size (170bp, 500bp, 2kb, 5kb, 10kb, 20kb) (Table1). The average depth of CHS was 71.87 ± 23.52, and that of CHB was 82.36 ± 14.13. The average genome coverage of CHS was 99.65% ± 0.34%, and that of CHB was 99.60% ± 0.30% (Table 2).

SNP/INDEL discovery

Read alignment was performed using the *aln* algorithm of BWA 0.6.1[15], using reads with insert size ranging from 180bp to 500bp, to align against the human genome (hg19/GRCh37). We used GATK (version 2.7.1) [16, 17] to remove duplications, realign around InDels, and recalibrate alignment quality scores. SNPs and InDels calling was performed using GATK UnifiedGenotyper. After VQSR filtering, we ultimately obtained 12,568,804 SNPs and 2,074,210 InDels. The step-by-step procedures and command lines of the variants calling have been generated using the platform of protocols.io (https://www.protocols.io/view/snp-indel-calling-grkbv4w). Of these variants, 12,536,571 SNPs and 1,472,925 InDels were bi-allelic, 4,573,367 were rare variants (MAF < =1%), 3,013,158 were low frequency (1% < MAF <= 5%), and 6,422,971 were common (MAF > 5%). Gene-based annotations for SNPs and InDels were also performed using ANNOVAR[18] based on the reference genome of Hg19. We found that 5,651,762 SNPs and 982,480 InDels were distributed in gene regions, respectively, and that more than half of the variants were found in the intergenic regions (Table 3).

Variation evaluation

To evaluate the SNP set, we first randomly selected 22 samples, and performed genotyping using the Illumina Infinium OmniZhongHua-8 v1 BeadChip. 407,040 ± 2,635 SNPs were obtained and the concordance rate was 99.94%±0.02% (False discovery rate (FDR), 0.06% ± 0.02%). We then compared our SNP set with several public genotype data sets, including Affymetrix Affy6.0, Illumina Omni2.5 arrays and the variation set of the 1000GP (phase III). For Affymetrix Affy6.0 and Illumina Omni2.5 arrays, we compared the variants of 86 individuals with genotyping overlap between the two platforms. As described in the comparison with Illumina Infinium OmniZhongHua-8 v1 BeadChip array, FDR rates were both below 0.1% (Table 4). Finally, we ran a comparison between our SNP set and the 1000GP (phase III) SNP set, and counted the SNP set overlap between these two sets, ennumerating the FDR to be 0.32%±0.07%. For InDels, we also compared two sets using the same method, for which the FDR was 2.72% ± 0.15% (Table 4).

In order to highlight the strengths of deep sequencing, and to mine potential novel variants particular to Han Chinese, we compared our variant set to the respective sets of the 1000GP, the Han Chinese subset

of the 1000GP, and to the dbSNP (build 147). The results showed that a large amount of novel variants were found in all three comparisons. In the comparison to the set of the Han Chinese the 1000GP, 13.17% SNPs and 43.99% InDels prove novel (Figure 1). Among these novel variants, over 85% of the SNPs and 51% InDels can be classified as low frequency or rare, and the proportion of rare variants was larger than that of low frequency variants. This feature of the variants was particularly visible in the comparison between our set and Han Chinese set of 1000GP, in which the low-frequency SNPs (MAF < 5%) account for 91.7% of novel set, and in which the rare variants are as high a fraction as 56.8% (Figure 2).

In summary, the deep genome sequencing of 90 Han Chinese individuals presents a powerful performance in characterizing novel variants, of which the majority are low frequency or rare. It also lays a foundation to explore the potential functions of these variants in further studies.

Genome assembly

We used SOAPdenovo2[19] to assemble the genome of each individual, based on libraries with hierarchical insert size. For the genomic data of each individual, we performed several analysis steps before genome assembly. These steps are as follows: 1) filtering of low quality reads, correcting of base calling errors; 2) filtering out reads with adapters (match length >= 10bp, mismatch <=3); 3) filtering out reads with fractions of N larger than 10%; 4) filtering out reads with low quality base rates in excess of 40%; 5) removal of duplicated reads produced in PCR amplification; 6) calculation of k-mer frequency of all reads, in order to generate frequency tables; and 7) removal of reads with low frequency k-mers. Detailed protocol information about *denovo* assembly has been described on the protocol.io (https://www.protocols.io/view/soapdenovo-genome-assembly-gr3bv8n).

We finally used reads with insert size below 2k to assemble the contigs, and used all reads to assemble scaffolds. The k-mer size of *denovo* assembly was set as 63, and the merge level was 2. Counting the assembled genomes, the average genome size was 2,951,301,058 ± 12,168,854 bps, the average contigs N50 was 2,865 ± 97 bps, and the average contig size was 49,339 ± 6,088 bps.

Structural variations calling and genotyping

We here applied an integrative strategy to identify the SVs of Han Chinese, including multiple current algorithms. The assembly-based method (SOAPsv) [26] was firstly employed in SV calling. We finally obtained a total of 26,142 SVs, containing 12,772 insertions and 13,370 deletions. The average number for each individual is 3102 ± 190. Besides this, several other methods were then applied to call SVs, including Pindel [20], CNVnator [21], Breakdancer [22], and Genome STRiP [23]. More detailed

protocol information on SV calling can be found on the protocols.io platform (https://www.protocols.io/view/structure-variation-detection-gr4bv8w). We then merged all of the deletions in several SV sets according to their breakpoints, to obtain an integrative deletion set. These deletions were genotyped in the tool Genome STRiP. We then discarded the SVs with large proportions of un-qualified genotypes (> 10%), which include genotypes of low quality (phred-scaled likelihood score below 13), and which were missing. After the above steps, a total of 24,369 deletions passed this filtering. After annotation by RepeatMasker[24], over 70% of these deletions were found to be in the simple repeat (31.23%), the Alu (23.52%), and the L1 repeat elements (15.66%) (Figure 3).

Length distribution showed an enrichment of short deletions (< 5 Kb), accounting for 76.77% of all deletions. Only 0.45% of these proved larger than 500 Kb. Based on the criterion of 50% reciprocal overlap [12], we found that 61.58% of our deletion set are novel with respect to the SVs of the 1000GP. 20% of the novel deletions are low frequency (MAF < 5%). We then evaluate the genotype concordance by comparing with the SV set of the 1000GP. We compared genotypes of the overlapping SVs in both sets. Concordance rates reached 89.45%±0.004%. It is as high as 94.90% when the proportion of reciprocal overlap is set at 85% (Figure 4).

We built a more comprehensive SV catalogue for Han Chinese using the above, integrative strategy, including assembly-based method, Pindel, CNVnator, Breakdancer and Genome STRiP. This catalogue harbors significantly more SVs than that of the Han Chinese from 1000GP (~7700), and the majority of them are novel (17,927, 73.56%). The set overlap in SVs between the two sets evidences high concordance of genotype. This provides valuable data panel for use in contributing to research into human diversity and genetic diseases.

Notes

Tianming Lan and Haoxiang Lin contributed equally to this work.

Abbreviations

1000GP, 1000 Genome Project; HRC, Haplotype Reference Consortium; NGS, Next Generation Sequencing; GWAS, Genome-Wide Association Study; MAF, Minor Allele Frequency; FDR, false discovery rate; CHB, Han Chinese in Beijing; CHS, Southern Han Chinese; LRT, Likelihood Ratio Test; GATK, Genome Analysis Tool Kit; VQSR, Variant Quality Score Recalibration.

Acknowledgements

We thank for the support of Shenzhen Municipal of Government of China (CXB201108250094A).

Availability of supporting data

The raw fastq format data were deposited at EBI with the project accession PRJEB11005, and the secondary accession ERP012319. The datasets in this article are available in the GigaDB database. VCFs have been archived at EMBL-EBI under accession PRJEB20820. All code and procedures concerning variants calling and assembly are accessible via https://github.com/HaoxiangLin/WGS_of_Han_Chinese_genomes. Datasets further supporting the results of this data note are available in the GigaScience Database[25].

Authors' contributions

XX, HY, JW, JW and XL conceived this project. HL and XG collected the samples, isolated the genomic DNA and constructed the DNA libraries. XG, TL, and WZ performed the genome analysis. LCAMT provided advice. XG and TL submitted the dataset to GigaDB. TL, XG and LCAMT wrote the article. All authors discuss the project and data. All authors read and approved the final manuscript.

Competing interests

The authors declare that they have no competing interests.

Author details

[1]BGI-Shenzhen, Shenzhen 518083, China. [2]Department of Biology, University of Copenhagen, Ole Maaløes Vej 5, 2200 Copenhagen, Denmark. [3]Shenzhen Key Laboratory of Neurogenomics, BGI-Shenzhen, Shenzhen 518083, China. [4]James D. Watson Institute of Genome Sciences, Hangzhou 310058, China

Reference

1. Genomes Project C, Abecasis GR, Altshuler D, Auton A, Brooks LD, Durbin RM, Gibbs RA, Hurles ME, McVean GA: **A map of human genome variation from population-scale sequencing**. *Nature* 2010, **467**(7319):1061-1073.
2. Genomes Project C, Abecasis GR, Auton A, Brooks LD, DePristo MA, Durbin RM, Handsaker RE, Kang HM, Marth GT, McVean GA: **An integrated map of genetic variation from 1,092 human genomes**. *Nature* 2012, **491**(7422):56-65.
3. Genomes Project C, Auton A, Brooks LD, Durbin RM, Garrison EP, Kang HM, Korbel JO, Marchini JL, McCarthy S, McVean GA *et al*: **A global reference for human genetic variation**. *Nature* 2015, **526**(7571):68-74.
4. McCarthy S, Das S, Kretzschmar W, Delaneau O, Wood AR, Teumer A, Kang HM, Fuchsberger C, Danecek P, Sharp K *et al*: **A reference panel of 64,976 haplotypes for genotype imputation**. *Nature genetics* 2016, **48**(10):1279-1283.
5. Li Y, Sidore C, Kang HM, Boehnke M, Abecasis GR: **Low-coverage sequencing: implications for design of complex trait association studies**. *Genome research* 2011, **21**(6):940-951.

6. Consortium UK, Walter K, Min JL, Huang J, Crooks L, Memari Y, McCarthy S, Perry JR, Xu C, Futema M *et al*: **The UK10K project identifies rare variants in health and disease**. *Nature* 2015, **526**(7571):82-90.
7. Bizon C, Spiegel M, Chasse SA, Gizer IR, Li Y, Malc EP, Mieczkowski PA, Sailsbery JK, Wang X, Ehlers CL *et al*: **Variant calling in low-coverage whole genome sequencing of a Native American population sample**. *BMC genomics* 2014, **15**:85.
8. Yi X, Liang Y, Huerta-Sanchez E, Jin X, Cuo ZX, Pool JE, Xu X, Jiang H, Vinckenbosch N, Korneliussen TS *et al*: **Sequencing of 50 human exomes reveals adaptation to high altitude**. *Science* 2010, **329**(5987):75-78.
9. Auer PL, Lettre G: **Rare variant association studies: considerations, challenges and opportunities**. *Genome medicine* 2015, **7**(1):16.
10. Feuk L, Carson AR, Scherer SW: **Structural variation in the human genome**. *Nature reviews Genetics* 2006, **7**(2):85-97.
11. Pang AW, MacDonald JR, Pinto D, Wei J, Rafiq MA, Conrad DF, Park H, Hurles ME, Lee C, Venter JC *et al*: **Towards a comprehensive structural variation map of an individual human genome**. *Genome biology* 2010, **11**(5):R52.
12. Sudmant PH, Rausch T, Gardner EJ, Handsaker RE, Abyzov A, Huddleston J, Zhang Y, Ye K, Jun G, Hsi-Yang Fritz M *et al*: **An integrated map of structural variation in 2,504 human genomes**. *Nature* 2015, **526**(7571):75-81.
13. English AC, Salerno WJ, Hampton OA, Gonzaga-Jauregui C, Ambreth S, Ritter DI, Beck CR, Davis CF, Dahdouli M, Ma S *et al*: **Assessing structural variation in a personal genome-towards a human reference diploid genome**. *BMC genomics* 2015, **16**:286.
14. Li Y, Zheng H, Luo R, Wu H, Zhu H, Li R, Cao H, Wu B, Huang S, Shao H *et al*: **Structural variation in two human genomes mapped at single-nucleotide resolution by whole genome de novo assembly**. *Nature biotechnology* 2011, **29**(8):723-730.
15. Li H, Durbin R: **Fast and accurate long-read alignment with Burrows-Wheeler transform**. *Bioinformatics* 2010, **26**(5):589-595.
16. McKenna A, Hanna M, Banks E, Sivachenko A, Cibulskis K, Kernytsky A, Garimella K, Altshuler D, Gabriel S, Daly M *et al*: **The Genome Analysis Toolkit: a MapReduce framework for analyzing next-generation DNA sequencing data**. *Genome research* 2010, **20**(9):1297-1303.
17. DePristo MA, Banks E, Poplin R, Garimella KV, Maguire JR, Hartl C, Philippakis AA, del Angel G, Rivas MA, Hanna M *et al*: **A framework for variation discovery and genotyping using next-generation DNA sequencing data**. *Nature genetics* 2011, **43**(5):491-498.
18. Wang K, Li M, Hakonarson H: **ANNOVAR: functional annotation of genetic variants from high-throughput sequencing data**. *Nucleic acids research* 2010, **38**(16):e164.
19. Luo R, Liu B, Xie Y, Li Z, Huang W, Yuan J, He G, Chen Y, Pan Q, Liu Y *et al*: **SOAPdenovo2: an empirically improved memory-efficient short-read de novo assembler**. *GigaScience* 2012, **1**(1):18.
20. Ye K, Schulz MH, Long Q, Apweiler R, Ning Z: **Pindel: a pattern growth approach to detect break points of large deletions and medium sized insertions from paired-end short reads**. *Bioinformatics* 2009, **25**(21):2865-2871.
21. Abyzov A, Urban AE, Snyder M, Gerstein M: **CNVnator: an approach to discover, genotype, and characterize typical and atypical CNVs from family and population genome sequencing**. *Genome research* 2011, **21**(6):974-984.
22. Chen K, Wallis JW, McLellan MD, Larson DE, Kalicki JM, Pohl CS, McGrath SD, Wendl MC, Zhang Q, Locke DP *et al*: **BreakDancer: an algorithm for high-resolution mapping of genomic structural variation**. *Nature methods* 2009, **6**(9):677-681.
23. Handsaker RE, Korn JM, Nemesh J, McCarroll SA: **Discovery and genotyping of genome**

structural polymorphism by sequencing on a population scale. *Nature genetics* 2011, **43**(3):269-276.

24. Saha S, Bridges S, Magbanua ZV, Peterson DG: **Computational Approaches and Tools Used in Identification of Dispersed Repetitive DNA Sequences**. *Tropical Plant Biology* 2008, **1**(1):85-96.

25. Lan T-M, Lin H-X, Zhu W-J, Tellier L-C-A-M, Yang M-C, Liu X, Wang J, Wang J, Yang H-M, Xu X, Guo X-S. Supporting data for **"Deep whole-genome sequencing of 90 Han Chinese genomes"**. GigaScience Database. 2017. http://dx.doi.org/xxxxxxxxx
