# Supplementary material for: Deep whole-genome sequencing of 90 Han Chinese genomes
Source: Gigascience. 2017 Jul 31;6(9):1–7. doi: 10.1093/gigascience/gix067 (PMC5603764; doi:10.1093/gigascience/gix067)
Supplement: Response-to-Reviewer-Comments_Original-Submission.pdf [file gix067_Response-to-Reviewer-Comments_Original-Submission.pdf]

Author's Response To Reviewer Comments

GIGA-D-16-00115

Deep whole-genome sequencing of 90 Han Chinese genomes
Tianming Lan*; Haoxiang Lin*; Wenjuan Zhu; Tellier Christian Asker Melchior Laurent; Xin Liu; Jun Wang; Jian Wang; Huanming Yang; Xun Xu; Xiaosen Guo
GigaScience

Dear Xiaosen,

Apologies for the delay, but we finally have the last review in for your paper. Your manuscript "Deep whole genome sequencing of 90 Han Chinese genomes" (GIGA-D-16-00115) has been assessed by our reviewers. We are unable to consider it for publication in its current form without some additional. The reviewers have raised a number of points which will improve the manuscript and if addressed sufficiently will allow a revised version to be published in GigaScience.

Their reports, together with any other comments, are below. Please also take a moment to check our website at http://giga.edmgr.com/ for any additional comments that were saved as attachments.

Q: As you'll see - a lot more information is needed to help clarify the source of this data and how exactly it relates to the 1000 genome project (is it previously unreleased genomes, or from the same individuals as the 1000 genomes).

A: Yes, we have clarified the source of the samples and the data in the revised manuscript. The samples in this study are the part of Han Chines of the 1000 Genomes Project and were purchased from the Coriell Institute for Medical Research. We performed high-coverage whole genome sequencing for each of them.

Q: You will all need to provide accessions for all the data and make sure the raw data is available and live in the NCBI/ENA SRA.

A: Yes, we have uploaded the raw data on the EBI, and we have provided the accession number for all raw genomic data (FASTQ). The access number has included in the main text.

Q: More methodological detail is also required. The protocols.io platform can assist in providing step-by-step protocol information if that helps (if you fill out SOPs in our GigaDB submission form we can help generate these protocols on your behalf).

A: Thank you for your super suggestion. The detailed methods of SNP/INDEL/SV calling and validation, the assembly process, and the comparisons with 1KG were added in the new version and the command lines also are supplied by using the protocols.io platform. The URLs of the protocols.io has been provided in the revised version.

Once you are able to fully address these points, we would ask you to submit a revised manuscript to GigaScience. Once you have made the necessary corrections, please submit online at: http://giga.edmgr.com/

If you have forgotten your username or password please use the "Send Login Details" link to get your login information. For security reasons, your password will be reset.

Please include a point-by-point within the 'Response to Reviewers' box in the submission system. ？Please ensure you describe additional experiments that were carried out and include a detailed rebuttal of any criticisms or requested revisions that you disagreed with. Please also ensure that your revised manuscript conforms to the journal style, which can be found in the Instructions for Authors on the journal homepage.

The due date for submitting the revised version of your article is 06 Mar 2017.
I look forward to receiving your revised manuscript soon.

Best wishes,
Hans Zauner
GigaScience
www.gigasciencejournal.com

Reviewer reports:
Reviewer #1: It is great to see that you have done deep sequencing on these 90 Chinese cell lines. Better genotypes for different populations will help build more accurate imputation panels for wide scale sequencing. If you intend to use this data as such for your own research though please do be aware of this recent preprint http://biorxiv.org/content/early/2016/10/13/080770 about combining population specific panels with the 1000 Genomes data.

I believe the article needs more information before it can be published.
Q: Table 3 in the publication has a column in dbSNP 135 - what does this refer to, does it mean the number of filtered sites which were found in dbSNP?

A: Yes, we check the table again and have canceled this table due to the ambiguity. We revised the process of genotype calling, and a new table has been given out in the revised manuscript. We applied a newer version of dbSNP (build 147) and updated related analyses in the revised manuscript.

Analysis process
Q: There is no record of command lines for the analyses run. What settings were giving to the alignment programs, variant callers or genotyping programs? How was VQSR uses, what filtering criteria. What about how the assemblies were created?
The description mentions some in house filtering without actually providing any pointers to either a fuller description of the process or the code uses to run the filtering for the genotyping process.

A: The command line for alignments, variant calling, genotyping and VQSR were all supplied in

the revised version, and we generated these processes using prococol.io platform.

Reference data

Q: What reference genome was used? very late in the document hg19 is mentioned but no pointer to an actual fasta file, did it include the 1000G decoy, why was hg19 used and not GRCh38

A: Yes, we used human genome (hg19/GRCh37) as the reference in our analyses. Since we performed the sequencing and related analyses one more years ago, the related annotation files of GRCh38 version were incomplete. We did not used the reference with the 1000G decoy included.

Raw data

Q: The FTP location I was given did not contain sequence or alignment data? has this been archives? if so where? in not why not? Variants and assemblies I was given an FTP site to look at the data. The connection was very poor so I was only able to peek at the files.

A: We have submitted the raw genomic data (FASTQ) to the EBI database. The accession number is PRJEB11005, and the secondary accession ERP012319. Given the alignment results can be obtained from raw data and they are so large (> 20 Tb), we did not upload them to EBI. About the poor connection, we will connect the journal office to solve it. Additionally, the raw data had been submitted to EBI, you can access them via the access numbers. We also will submit the variants and assembly to the database.

Q: The files themselves are named very poorly SNP.vcf.gz doesn't give the user any information about what the file contains

A: Thank you for your comments. We have revised the file name and added more detailed information in the new uploaded files.

Q: The SNP vcf file has many stats in the genotype column without any information about how to interpret those statistics

A: These interpretation has been listed at the header of the vcf file.
AD, Allelic depths for the ref and alt alleles in the order listed.
DP, Approximate read depth (reads with MQ=255 or with bad mates are filtered.
GQ, Genotype Quality.
GT, Genotype.
PL, Normalized, Phred-scaled likelihoods for genotypes as defined in the VCF specification.

Q: The SV vcf did not contain any individual genotypes. Neither of the README files contain any information about how the process was run, just the same light detail free description as the main paper

A: We have generated a new SV set which contains detailed genotype information of all 90 individuals. We also rewrite the process of SV calling and genotyping.

Q: What is the archive plan for this data? how is it going to be released to the public?

A: We have uploaded the raw data to the EBI, and the other variation data, including SNPs, INDELs and SVs, will be saved at the FTP sites the gigascience supplied. All data will be released to the public while the paper published.

I am happy to talk to the authors about these issues. Having managed the 1000 Genomes data I have a good feel for best practice in this field.thanks
Laura

Reviewer #2: The study by Lan and Lin et al. provides high-depth sequencing data from 90 Chinese genomes. It is of interest to the human genomics community, especially those who are interested in rare variants, genomic structural variants, and de novo genome assemblies. The dataset is especially relevant since the authors sequenced multiple libraries with different insert sizes. This will allow researchers to construct integrative approaches for resolving complex regions of the genome and structural variants within them. Overall, the study is relevant to the field and the dataset provided here will be used by researchers with different interests. Having said these, before publication, some important points need to be addressed:
1. It is my understanding that the samples that are sequenced here are the same as the Chinese samples sequenced by 1000 Genomes Project. This needs to be made clear in the abstract. Also, from various parts of the text read as if the authors collected the samples and/or secured the consent forms themselves. I am assuming that this is not the case and it should be clarified both throughout the main text, methods and abstract.

A: Yes, the samples in this study are the part of Han Chinese of the 1000 Genomes Project and 83 of them were sequenced by the project. We have clarified the detailed information in the revised manuscript, including the abstract and main text.

2. In the abstract, and elsewhere in the text, there are implications that Chinese population is special with regards to the need for improved sequencing. This needs to be either toned down or explained better. For example, in the abstract it is stated that ".. data is not always sufficient for this purpose, especially for Chinese populations"... Why?

A: Yes, we agree with you. We have re-written the related parts and we think important benefits of this Han Chinese data should be a valuable supplement for the 1000 Genomes Project and other human genome plan, especially for the discovery of low frequency variants of Han Chinese.

3. The statements in the Background with regards to the accuracy of 1000 Genomes dataset is not accurately portrayed. To my knowledge, 1000 Genomes Project do not use standard variant calling tools on individual low-coverage genomes. Instead, they utilized custom variant calling pipelines integrating data from thousands of genomes. As such, the variant calls are highly accurate. It is, however, possible that some of the rare variants have been filtered out and there may be a good number of false-negatives within 1000 Genomes Phase 3 datasets. This paper would be able to catch those for the Chinese population. As such, the emphasis should be on

calling rare variants that may have been missed by 1000 Genomes.

A: You are right. The variant calls of the 1000 Genomes Project are high-quality, the expression in our previous manuscript is somewhat inappropriate. We have added some comparative analyses between our variant sets and the 1000 Genomes Project and re-written the manuscript. From the comparison, we found majority novel variants were low frequency and rare. In our revised version, we focus on the identification of low frequency and rare variants that may have been missed in the 1000 Genomes Project.

4. Given that these samples have already been sequenced by 1000 Genomes, I am confused as to how additional regional characteristics would be captured by this study as it is claimed. This part should be rewritten as it is currently misleading.

A: Thank you for your comments. As we know, the variants call sets were obtained based on low-coverage whole-genome sequencing (WGS) and partial deep sequencing (whole exome sequencing, WES). Large proportion of genomic regions is only covered by low-coverage data, which probably miss a certain amount of variants, especially the low-frequency and rare sites. We here performed the deep sequencing of the Han Chinese samples to identify those potential variants, especially the SVs. The related expressions were inappropriate, and we here have modified them in our revised version.

5. The strength of this dataset, in my opinion, is not necessarily identifying new single nucleotide variants, but to identify structural variants that may have been missed by 1000 Genomes. I would emphasize this section and add a paragraph describing this aspect of the dataset in the background.

A: Yes, you are right. Besides some new analyses of SNPs and indels, we also added the comparative analyses of SVs between our set and that of the 1000 Genomes Project. We also present some new results in our revised version.

6. Even though this is a data report, I think it is required to have a comparison of the variant calls in this dataset with the 1000 Genomes variant calls from the same samples. This is important given that these samples have already been sequenced before and the novelty of this new dataset depends on how many novel variations that it will determine. As such, it would be helpful to see a basic comparison of the single nucleotide, insertion deletion and structural variants that were found in this study and 1000 Genomes for these samples.

A: Yes, we made a comprehensive comparison between our data set and the 1000 Genomes Project, including SNPs, InDels and SVs. The detailed results and methods could be found in our revised manuscript. Besides, we also compared our data set with dbSNP.

7. The language needs to be tightened. I understand everything that the authors wrote. However, there are multiple instances of grammatical errors or unclear wording. As such, a careful reading and editing would be essential before publication.
For example:
- Abstract: line 26: has been primarily -> has primarily been

- Abstract: line 49: low pass sequencing -> low coverage sequencing
- Background: Backgrounds -> Background
- Background Line 8: -> NGS -> Next generation sequencing (NGS)
- Background Line 17: tens thousands -> tens of thousands

A: Thanks for your suggestions for the languages tightening, and we have invited a native speaker for English to polish our revised manuscript.

Reviewer #3: The authors make available an important resource, deep whole genome sequencing data of 90 Han Chinese samples. Since the majority of sequencing data generated so far has been on individuals of European descent this resource could potentially be of great use to other researchers working with genomic data from the Han Chinese population.
Please find my comments below.
1. In the background section the authors state "each sample from the 1000 Genomes Project has an average sequencing depth of only 5~7X, which is low that heterozygous variants are frequently incorrectly called, and false variants are commonly introduced." Could the authors cite a manuscript to support this claim? If the authors are speculating here the tone of the sentence could be modified accordingly since the overall goals of the 1000 Genomes Project were different from this project.

A: Yes, you are right. The variant calls of the 1000 Genomes Project are high quality. The related expressions in our previous version are somewhat inappropriate. We have re-written related descriptions. We also make comparison with the 1000 Genomes Project and found that the majority of the novel variants were low frequency and rare. The emphasis in our revised version is on calling low frequency and rare variants that may have been missed in the 1000 Genomes Project.

2. "The 1000 Genomes data is not suitable imputation panel for specifically Chinese populations, due to regional genetic characteristics of the samples gathered for the panel." This sentence is a little unclear, could the authors elaborate? Since the samples sequenced by this project are the same as the 1000 Genomes wouldn't this data-set lead to similar results?

A: Thank you for your comments. Yes, our expressions are somewhat inappropriate and we have modified them in out revised version. As we know, the variants call sets were obtained based on low-coverage whole-genome sequencing (WGS) and partial deep sequencing (whole exome sequencing, WES). Large proportion of genomic regions is only covered by low-coverage data, which probably miss a certain amount of variants, especially the low-frequency and rare sites. We here performed the deep sequencing of the Han Chinese samples to identify those potential variants, especially the SVs.

3. In the SNP/INDEL discovery section, the authors describe the number of common, low-frequency and rare variants, could the authors mention the allele frequency cutoffs for these three classes?

A: Thank you for your advice. We have added the cutoffs for these classes in our revised version. In this study, we defined the minor allele frequency (MAF) of <1%, from 1% to 5% and

>5% as the rare, low-frequency and common variants respectively.

4. The authors provide the annotation for the deletion SVs. Could the authors provide an average breakdown by SV type (number of duplications, deletions, translocations etc)?

A: To obtain the high-confidence SVs, we used an integrative SV calling strategy, which including multiple methods of assembly-based method, Pindel, Breakdancer, and so on. We finally integrated multiple SV sets and only kept the high-confidence deletions. The statistics and comparisons with that of the 1000 Genomes Project were all presented in our revised manuscript.

5. Have the authors compared their SNP/INDEL call-sets to the results from the 1000 Genomes Project. This could be helpful in two ways a.) Discover any systematic errors if any in their variant-calling approach. b) Illustrate the power of this analysis/data over the 1000 Genomes dataset.

A: Yes, you are right, the comparative analyses will make sense for the further studies, such as assessing the methods, characterizing the potential functional variants, and so on. We here made a comprehensive comparison between our call sets and the 1000 Genomes Project, including SNPs, Indels and SVs. The detailed results and methods could be found in our revised manuscript.

6. The authors specify the advantages of this dataset for genotype imputation, I'm curious to hear if the authors have taken a stab at this using this dataset for the same. Would 90 high-depth samples be sufficient for this? This analysis is of-course not critical to this data-note.

A: You are right. Our description in the previous version is inappropriate and we have re-written related parts. As we know, we can identify much more potential variants through deep sequencing that may miss based on low-coverage sequencing, especially the SVs from assembly-based method. As expected, majority of the novel sites are low-frequency or rare.

7. The authors include a genotype imputation step in their genotype calling steps, could the authors indicate which reference panel was used for this step and mention if this choice might affect their genotype calling results?

A: We have been aware that the description of imputation is inappropriate and have modified related parts in the revised manuscript.

8. Would it be possible to also include brief annotations of the SNVs and INDELs since the authors seem to do this for the deletion SVs?

A: Yes, we have added the gene-based annotation of SNPs and INDELs in revised version.

9. Could the authors indicate the samples from which the SV was called (perhaps in the INFO field) in the SV genotypes VCF file?

A: Yes, we have offered two SV data sets in the revised manuscript, the sample information

could be found in any of the two variants sets. These two sets will be soon uploaded to the FTP site.

10. Minor grammatical errors
- "Background" instead of "Backgrounds" in the section title
- "Two stages were involved in SV calling: the first phase is fast mapping, which using the BWA-SW to locate contigs in the reference genome. The second phase is the LASTZ aligner, to hold large gaps on the alignments."
- "High-depth sequencing of these samples has supplied us with highly confident variant calls upon the samples"

A: Thanks for your suggestions for the languages tightening, and we have invited a native speaker for English to polish our revised manuscript.

Reviewer #4: Sequencing additional populations is of importance for many genetic studies, particularly the GWAS/imputation approaches discussed in the introduction. The data produced by the current study, and the methods used to produce it, appears to be of high quality. However, I feel several points must be addressed to evaluate the extent to which this is an advance for the field.
(a) The authors state that the 90 sequenced samples are already a part of the 1000G project. Thus, their point in the introduction that "the 1000 Genomes data is not suitable imputation panel for specifically Chinese populations" did not make sense to me. How is this work addressing this limitation, if no new samples have been sequenced?

A: Yes, we agree with you. We have make a comparison between the 1000 Genome Project and some Asian populations concerning the power of genotype imputation (unpublished). The results showed that the data of the 1000 Genomes Project was good enough for imputations, including the Han Chinese population. So, we have modified the related description in out revised version.

(b) If no new samples have been sequenced, then the main difference between the presented data and 1000G is the deep sequencing. Could the authors add a comparison as to how much is gained relative to 1000G as a result? Usually low-coverage sequencing is pretty good down to 0.5% frequency or so, below which imputation doesn't really work anyway. Thus the authors need to address how much is really gained from this study, ideally through a comparison to 1000G and with more detail of the types of variants that can now be studied/imputed better as a result.

A: Yes, as your suggestion, we made a comprehensive comparison between our data set and the 1000 Genome Project, including SNPs, InDels and SVs. We found ~12% novel SNPs, 41% novel INDELs and 61.83% novel SVs. We also compared the overlapped sites with 1000GP, and we got a high concordance rate. The detailed results and methods could be found in our revised manuscript.

(c) With imputation used as the motivating factor, the authors need to reference the HRC (http://biorxiv.org/content/early/2015/12/23/035170) and ideally reference how their work compares to it, rather than just 1000G. HRC is predominantly European but it should be mentioned.

A: Yes, HRC is a very valuable and useful reference panel, and we have referred this project in our abstract and the main text.

(d) In general I'd like to see a few figures/tables showing the technical quality of the data. Do we have any confidence that the variants are accurate? How well did indel calling work? How about SVs? There are standard metrics for assessing this so it would be good to include some of them. In particular, seeing how these compared to other published efforts such as 1000G would be good.

A: Yes, as your questions in (b), we compared the overlapped sites with the 1000 Genome Project. We got a high concordance rate for SNPs (>99.79%), INDELs (97.44%) and SVs (94.90%). We also randomly selected 22 samples and performed the genotyping using the Illumina OmniZhongHua-8 chip, 407,040 SNPs were obtained and the concordance rate was 99.94%. The detailed information about this part has been recorded in the revised manuscript.
--
Please also take a moment to check our website at for any additional comments that were saved as attachments. Please note that as GigaScience has a policy of open peer review, you will be able to see the names of the reviewers.
