# Supplementary material for: Deep whole-genome sequencing of 90 Han Chinese genomes
Source: Gigascience. 2017 Jul 31;6(9):1–7. doi: 10.1093/gigascience/gix067 (PMC5603764; doi:10.1093/gigascience/gix067)
Supplement: Response-to-Reviewer-Comments_Revision-1.pdf [file gix067_Response-to-Reviewer-Comments_Revision-1.pdf]

Author's Response To Reviewer Comments

Date: 10 Apr 2017
To: "Xiaosen Guo" guoxs@genomics.cn
From: "GigaScience Editorial Office" editorial@gigasciencejournal.com
Subject: Your submission to GigaScience - GIGA-D-16-00115R1
GIGA-D-16-00115R1
Deep whole-genome sequencing of 90 Han Chinese genomes
Tianming Lan; Haoxiang Lin; Wenjuan Zhu; Tellier Christian Asker Melchior Laurent; Mengcheng Yang; Xin Liu; Jun Wang; Jian Wang; Huanming Yang; Xun Xu; Xiaosen Guo
GigaScience

Dear Mr Guo,

Your revised manuscript "Deep whole-genome sequencing of 90 Han Chinese genomes" (GIGA-D-16-00115R1) has been re-reviewed. Based on the latest reports, and my own assessment as Editor, I am pleased to inform you that it is potentially acceptable for publication in GigaScience.

However, before we proceed, please address some additional recommendations from reviewer 1 (see below). The reviewer also has some useful further suggestions regarding sharing your variant data. Prior to acceptance, our in-house data curator will have a look over your data, and if you have any questions related to data sharing please do get in touch.

Their reports, together with any other comments, are below. Please also take a moment to check our website at http://giga.edmgr.com/ for any additional comments that were saved as attachments.

Once you have made the necessary corrections, please submit a revised manuscript online at:

http://giga.edmgr.com/

If you have forgotten your username or password please use the "Send Login Details" link to get your login information. For security reasons, your password will be reset.

Please include a point-by-point within the 'Response to Reviewers' box in the submission system. Please ensure you describe additional experiments that were carried out and include a detailed rebuttal of any criticisms or requested revisions that you disagreed with. Please also ensure that your revised manuscript conforms to the journal style, which can be found in the Instructions for Authors on the journal homepage.

The due date for submitting the revised version of your article is 09 Jul 2017.

We look forward to receiving your revised manuscript soon.

Best wishes,

Hans Zauner
GigaScience
www.gigasciencejournal.com

Reviewer reports:
Reviewer #1: Thank you for the improvements to the paper. I still have some small concerns.

As other reviewers noted in the first review. The paper seems to suggest the differences between the 1000 Genomes call set based on low coverage and exome data and the Han Chinese data is due to errors in the 1000 Genomes call set. It would be good to see it clearly stated that this was due to false negatives which is an understood issue for rare variant identification with low coverage data.

Q: Table 5 and Figure 1 aren't very clear in their explanation on the difference between rare variants in the high coverage Han Chinese set and the 1000 genomes sets. I wonder if this would be better represented as a venn or euler diagram with only 3 sets to consider (if you do one for snp and one for indel) would make it much clearer what was in both sets and what was only found in the Han chinese set.

A: Yes, we have revised Table 5 and Figure 1. For Table 5, we removed the table and created a group of venn, which might illustrated the results of novel variants clearly. For Figure 1, we also created a figure of histogram instead of the line chart.
As your suggestions, we think the combination of these two new figure can clearly showed the results of the difference between rare variants in the high coverage Han Chinese set and the 1000 genomes sets.

Q: It is good to see the raw sequencing data in the nucleotide archive. The variants should be submitted to the European Variation Archive (http://www.ebi.ac.uk/eva/). The authors should consider compressing the BAMs with CRAM and submitting them to the ENA too so the full chain of evidence is publicly available in a standard resource. The variants going into the EVA will also mean the novel variants are assigned rs numbers and get properly integrated into dbSNP and Ensembl.

A: Yes, we have submitted our VCFs to the European Variation Archive under accession PRJEB20820. For the BAMs, unfortunately, they have been canceled from our computer cluster. We removed the BAMs with two main reasons: a) we have validated the variants and we have obtained a reliable results; b) they took up too much storage space (~12 Tb). We hope to have your understanding. Thanks!

Q: Several places in the manuscript the chipname OmniZhongHua-8 is used as a name for the genotype chip used. If this is a custom chip design, that should be made clear. If it is a standard chip design, please use the illumina name for the chip
A: Yes, it is a standard chip design, the name in the manuscript have been standardized. Its name is Illumina Infinium OmniZhongHua-8 v1 BeadChip.

Q: Manuscript page 1 Line 50: 83 of these 90 have not been sequenced by the 1000 Genomes Project
Should this be 7 of these 90 have not been sequenced by the 1000 Genomes Project
A: Yes, thank you and you are right. We have revised this sentence.
Q: Manuscript page 3 line 40: All Samples used in this study where obtained from 1000GP
The correct statement here is that
All Samples used in this study were obtained from the 1000 Genomes Project cell line collection at the Coriell Cell line repository
A: Yes, we have revised this sentence.
Q: Manuscript page 4 line 33 Tabel 3 should be Table 3
A: Yes, thank you very much.
Q: Manuscript page 5 line 61 It mentioned protocols.io without any link to the specific documents. This needs to be a link
A: Yes, we have added the link to the protocols.io.
Q: Table 2. I am confused by the usage of the fraction of target covered statistic. This is general used for exome data and is not considered relevant for whole genome data as there are no targets to be covered.
A: Yes, we think your suggestion is right, and we have removed the fraction of target covered statistic from our manuscript. Thanks for your recommendation.
Q: From a style perspective, the pdfs for command lines are difficult to read, interpret and reuse. I would strongly advocate for the authors creating a public git repo or equivalent which gives text files and direct urls to link to the programs run and the versions of those programs used. This is much better from a reproducibility point of view.
A: As your suggestion, we have created a public git repo, and all code and procedures concerning variants calling and assembly are uploaded. They are accessible via https://github.com/HaoxiangLin/WGS_of_Han_Chinese_genomes.

Thanks

Laura Clarke

Reviewer #2: I commend the authors for detailed replies for my comments. I have no further issues with the manuscript. It would be a nice addition to the high-quality genome datasets that we have.

1. It is my understanding that the samples that are sequenced here are the same as the Chinese samples sequenced by 1000 Genomes Project. This needs to be made clear in the abstract. Also, from various parts of the text read as if the authors collected the samples and/or secured the consent forms themselves. I am assuming that this is not the case and it should be clarified both throughout the main text, methods and abstract.

A: Yes, All Samples used in this study were obtained from the 1000 Genomes Project cell line collection at the Coriell Cell line repository. We randomly selected 90 samples and sequenced all these samples with an average depth of ~80x. 83 of them were also sequenced by the 1000GP,

and these can be found in the paper of Phase III. 7 of these 90 samples were not sequenced by 1000GP but we did. We have clarified the detailed information in the revised manuscript, including the abstract and main text.

2. In the abstract, and elsewhere in the text, there are implications that Chinese population is special with regards to the need for improved sequencing. This needs to be either toned down or explained better. For example, in the abstract it is stated that ".. data is not always sufficient for this purpose, especially for Chinese populations"... Why?

A: Yes, we agree with you. We have toned down that Chinese population is special with regards to the need for improved sequencing. 1000GP has sequenced most of the samples that we collected in this study. Though the sequencing depth in 1000GP is only 5-7X, this data can also represent most of the feature of Chinese genetic structure. So we have re-written the related parts and we think important benefits of this Han Chinese data should be a valuable supplement for the 1000 Genomes Project and other human genome plan, especially for the discovery of low frequency variants of Han Chinese. As we expected, we did found ~10% novel variants and most of these novel variants were low frequency and rare.

3. The statements in the Background with regards to the accuracy of 1000 Genomes dataset is not accurately portrayed. To my knowledge, 1000 Genomes Project do not use standard variant calling tools on individual low-coverage genomes. Instead, they utilized custom variant calling pipelines integrating data from thousands of genomes. As such, the variant calls are highly accurate. It is, however, possible that some of the rare variants have been filtered out and there may be a good number of false-negatives within 1000 Genomes Phase 3 datasets. This paper would be able to catch those for the Chinese population. As such, the emphasis should be on calling rare variants that may have been missed by 1000 Genomes.

A: You are right. The variant calls of the 1000 Genomes Project are high-quality, the expression in our previous manuscript is somewhat inappropriate. We have added some comparative analyses between our variant sets and the 1000 Genomes Project and re-written the manuscript. We found that more than 99% SNPs and more than 97% indels in our VCFs were consistent with that in 1000 GP, which also proved that the variant set in of 1000GP are high quality. As your suggestions and expected, we found majority novel variants were low frequency and rare (More than 50%). In our revised version, we focus on the identification of low frequency and rare variants that may have been missed in the 1000 Genomes Project.

4. Given that these samples have already been sequenced by 1000 Genomes, I am confused as to how additional regional characteristics would be captured by this study as it is claimed. This part should be rewritten as it is currently misleading.

A: Thank you for your comments. As we know, the variants call sets in 1000GP were obtained based on low-coverage whole-genome sequencing (WGS) and partial deep sequencing (whole exome sequencing, WES). Large proportion of genomic regions is only covered by low-coverage data, which probably miss a certain amount of variants (false negative), especially the low-frequency and rare sites. We here performed the deep sequencing of the Han Chinese samples to identify those potential variants, especially the SVs. As we expected, we found many novel

variants, including SNPs, InDels, and SVs. Among these novel variants, most are low frequency and are. The related expressions were inappropriate, and we here have modified them in our revised version.

5. The strength of this dataset, in my opinion, is not necessarily identifying new single nucleotide variants, but to identify structural variants that may have been missed by 1000 Genomes. I would emphasize this section and add a paragraph describing this aspect of the dataset in the background.

A: Yes, you are right. There must be some missing SNPs and InDels in the VCFs of 1000GP, which was due to false negatives. This is an understood issue for rare variant identification with low coverage data. For SVs, however, we assembly all the 90 samples for the detection of SVs. So we think we should find more new and reliable SVs in our data set. In this revised manuscript, besides some new analyses of SNPs and indels, we added the comparative analyses of SVs between our set and that of the 1000 Genomes Project. We found many new SVs that not in the 1000GP, and most of these are low frequency. For the comparisons, we add a paragraph to present some new results in our revised version.

6. Even though this is a data report, I think it is required to have a comparison of the variant calls in this dataset with the 1000 Genomes variant calls from the same samples. This is important given that these samples have already been sequenced before and the novelty of this new dataset depends on how many novel variations that it will determine. As such, it would be helpful to see a basic comparison of the single nucleotide, insertion deletion and structural variants that were found in this study and 1000 Genomes for these samples.

A: Yes, we made a comprehensive comparison between our data set and the 1000 Genomes Project, including SNPs, InDels and SVs. We found the novel variants from the comparisons and we also analyzed the pattern and distribution against frequency of these novel variations. 2 main comparison were made: HAN Chinese from 1000GP and our data set; the complete data set in 1000GP and our data set. A detailed comparison and the method we use could be found in our revised manuscript. Besides, we also compared our data set with dbSNP, and we used the version of build 147.

7. The language needs to be tightened. I understand everything that the authors wrote. However, there are multiple instances of grammatical errors or unclear wording. As such, a careful reading and editing would be essential before publication.
For example:
- Abstract: line 26: has been primarily -> has primarily been
- Abstract: line 49: low pass sequencing -> low coverage sequencing
- Background: Backgrounds -> Background
- Background Line 8: -> NGS -> Next generation sequencing (NGS)
- Background Line 17: tens thousands -> tens of thousands

A: Thanks for your suggestions for the languages tightening, and we have invited a native speaker for English to polish our revised manuscript.

--
Please also take a moment to check our website at for any additional comments that were saved as attachments. Please note that as GigaScience has a policy of open peer review, you will be able to see the names of the reviewers.
