# Supplementary material for: Deep whole-genome sequencing of 90 Han Chinese genomes
Source: Gigascience. 2017 Jul 31;6(9):1–7. doi: 10.1093/gigascience/gix067 (PMC5603764; doi:10.1093/gigascience/gix067)
Supplement: Response-to-Reviewer-Comments_Revision-2.pdf [file gix067_Response-to-Reviewer-Comments_Revision-2.pdf]

Author's Response To Reviewer Comments

GIGA-D-16-00115R2
Deep whole-genome sequencing of 90 Han Chinese genomes
Tianming Lan; Haoxiang Lin; Wenjuan Zhu; Tellier Christian Asker Melchior Laurent; Mengcheng Yang; Xin Liu; Jun Wang; Jian Wang; Huanming Yang; Xun Xu; Xiaosen Guo
GigaScience

Dear Dr Guo,

Many thanks for submitting your latest revised manuscript. Overall, we are happy that you have carefully addressed the remaining concerns of the reviewer and we very much appreciate your efforts to make data and code available.

Q: One issue though that we feel is indeed problematic, considering Gigascience's strong committment to open data, is the absence of the BAM files. We do understand that you no longer have these and it may not be realistic to re-create the entire lost dataset, because of time constraints. And we also appreciate that the raw data as well as the end result (the VCFs) are available.
However, after discussing the issue with the editorial team, we feel that the entire procedure from raw data via BAM files to VCFs should be at least documented with some examples, for the sake of reproducibility. That is, we kindly ask you to recreate at least a subset of the alignments / BAM files and document the workflow in detail, with these re-created files as an example for how it was done.
I hope you agree this will improve the reproducibility of your work.

A: Yes, you are right. We absolutely agree this. We have generated the BAM files of NA18547 in triplicate and uploaded these files to the FTP you provided. We think this will largely improve the reproducibility of our works. Thanks again for your nice suggestions.

Q: On a minor note, when revising your submission, please also include a reference to the GigaDB entry to your list of citations, using the following format as an example:
[xx] Zheng L-Y, Guo X-S, He B, Sun L-J, Peng Y, Dong S-S, et al. Supporting data for "Title of your manuscript". GigaScience Database. 2017. http://dx.doi.org/xxxxxxxxx
Please cite this reference where appropriate in the paper (data availability and methods section, where appropriate). We will then add the final doi of the GigaDB entry prior to publication.

A: OK! We have include a reference to GigaDB entry to our list of citations using the example you give us. Thank you!

Once you have made the necessary corrections, please submit a revised manuscript online at:

http://giga.edmgr.com/

If you have forgotten your username or password please use the "Send Login Details" link to get your login information. For security reasons, your password will be reset.

We look forward to receiving your revised manuscript soon.

Best wishes,

Hans Zauner
GigaScience
www.gigasciencejournal.com
