# Supplementary material for: Deep whole-genome sequencing of 90 Han Chinese genomes
Source: Gigascience. 2017 Jul 31;6(9):1–7. doi: 10.1093/gigascience/gix067 (PMC5603764; doi:10.1093/gigascience/gix067)
Supplement: Response-to-Reviewer-Comments_Revision-3.pdf [file gix067_Response-to-Reviewer-Comments_Revision-3.pdf]

Author's Response To Reviewer Comments

GIGA-D-16-00115R3
Deep whole-genome sequencing of 90 Han Chinese genomes
Tianming Lan; Haoxiang Lin; Wenjuan Zhu; Tellier Christian Asker Melchior Laurent; Mengcheng Yang; Xin Liu; Jun Wang; Jian Wang; Huanming Yang; Xun Xu; Xiaosen Guo
GigaScience
GIGA-D-16-00115R3
Deep whole-genome sequencing of 90 Han Chinese genomes
Tianming Lan; Haoxiang Lin; Wenjuan Zhu; Tellier Christian Asker Melchior Laurent; Mengcheng Yang; Xin Liu; Jun Wang; Jian Wang; Huanming Yang; Xun Xu; Xiaosen Guo
GigaScience

Dear Mr Guo,

Thank you for letting us know that you wish to make minor corrections to your figure legends.

You can upload the revised version via the link below.

When making these corrections, please also add the full name (including name of the university / institution) of the institutional review board that approved the study.

Q: Please also add the doi of the GigaDB entry (http://dx.doi.org/10.5524/100302) to reference [25].
A: Yes, we have added this doi and revised the figure legends. Thanks!

Once you have made all necessary corrections, please submit a revised manuscript online at:

http://giga.edmgr.com/

If you have forgotten your username or password please use the "Send Login Details" link to get your login information. For security reasons, your password will be reset.

We look forward to receiving your revised manuscript soon.

Best wishes,

Hans Zauner
GigaScience
www.gigasciencejournal.com
