# Supplementary material for: Deep whole-genome sequencing of 90 Han Chinese genomes
Source: Gigascience. 2017 Jul 31;6(9):1–7. doi: 10.1093/gigascience/gix067 (PMC5603764; doi:10.1093/gigascience/gix067)
Supplement: Reviewer-1-Report-(Original-Submission).pdf [file gix067_Reviewer-1-Report-(Original-Submission).pdf]

Reviewer Report

Title: Deep whole-genome sequencing of 90 Han Chinese genomes

Version: Original Submission **Date:** 10/28/2016

Reviewer name: Laura Clarke

Reviewer Comments to Author:

It is great to see that you have done deep sequencing on these 90 Chinese cell lines. Better genotypes for different populations will help build more accurate imputation panels for wide scale sequencing. If you intend to use this data as such for your own research though please do be aware of this recent preprint http://biorxiv.org/content/early/2016/10/13/080770 about combining population specific panels with the 1000 Genomes data.I believe the article needs more information before it can be published.Table 3 in the publication has a column in dbSNP 135 - what does this refer to, does it mean the number of filtered sites which were found in dbSNP?Analysis processThere is no record of command lines for the analyses run. What settings were giving to the alignment programs, variant callers or genotyping programs? How was VQSR uses, what filtering criteria. What about how the assemblies were created?The description mentions some in house filtering without actually providing any pointers to either a fuller description of the process or the code uses to run the filtering for the genotyping process.Reference dataWhat reference genome was used? very late in the document hg19 is mentioned but no pointer to an actual fasta file, did it include the 1000G decoy, why was hg19 used and not GRCh38Raw dataThe FTP location I was given did not contain sequence or alignment data? has this been archives? if so where? in not why not?Variants and assembliesI was given an FTP site to look at the data. The connection was very poor so I was only able to peek at the filesThe files themselves are named very poorly SNP.vcf.gz doesn't give the user any information about what the file containsThe SNP vcf file has many stats in the genotype column without any information about how to interpret those statisticsThe SV vcf did not contain any individual genotypesNeither of the README files contain any information about how the process was run, just the same light detail free description as the main paperWhat is the archive plan for this data? how is it going to be released to the public?I am happy to talk to the authors about these issues. Having managed the 1000 Genomes data I have a good feel for best practice in this field.thanksLaura

Level of Interest

Please indicate how interesting you found the manuscript: An article of importance in its field

Quality of Written English

Please indicate the quality of language in the manuscript: Acceptable

Declaration of Competing Interests

Please complete a declaration of competing interests, considering the following questions:

- Have you in the past five years received reimbursements, fees, funding, or salary from an organisation that may in any way gain or lose financially from the publication of this manuscript, either now or in the future?
- Do you hold any stocks or shares in an organisation that may in any way gain or lose financially from the publication of this manuscript, either now or in the future?
- Do you hold or are you currently applying for any patents relating to the content of the manuscript?
- Have you received reimbursements, fees, funding, or salary from an organization that holds or has applied for patents relating to the content of the manuscript?
- Do you have any other financial competing interests?
- Do you have any non-financial competing interests in relation to this paper?

If you can answer no to all of the above, write 'I declare that I have no competing interests' below. If your reply is yes to any, please give details below.

I declare that I have no competing interests

I agree to the open peer review policy of the journal. I understand that my name will be included on my report to the authors and, if the manuscript is accepted for publication, my named report including any attachments I upload will be posted on the website along with the authors' responses. I agree for my report to be made available under an Open Access Creative Commons CC-BY license (http://creativecommons.org/licenses/by/4.0/). I understand that any comments which I do not wish to be included in my named report can be included as confidential comments to the editors, which will not be published.

I agree to the open peer review policy of the journal
