# Supplementary material for: Deep whole-genome sequencing of 90 Han Chinese genomes
Source: Gigascience. 2017 Jul 31;6(9):1–7. doi: 10.1093/gigascience/gix067 (PMC5603764; doi:10.1093/gigascience/gix067)
Supplement: Reviewer-1-Report-(Revision-1).pdf [file gix067_Reviewer-1-Report-(Revision-1).pdf]

Reviewer Report

Title: Deep whole-genome sequencing of 90 Han Chinese genomes

Version: Revision 1 **Date:** 3/26/2017

Reviewer name: Laura Clarke

Reviewer Comments to Author:

Thank you for the improvements to the paper. I still have some small concerns.

As other reviewers noted in the first review. The paper seems to suggest the differences between the 1000 Genomes call set based on low coverage and exome data and the Han Chinese data is due to errors in the 1000 Genomes call set. It would be good to see it clearly stated that this was due to false negatives which is an understood issue for rare variant identification with low coverage data.

Table 5 and Figure 1 aren't very clear in their explanation on the difference between rare variants in the high coverage Han Chinese set and the 1000 genomes sets. I wonder if this would be better represented as a venn or euler diagram with only 3 sets to consider (if you do one for snp and one for indel) would make it much clearer what was in both sets and what was only found in the Han chinese set.

It is good to see the raw sequencing data in the nucleotide archive. The variants should be submitted to the European Variation Archive (http://www.ebi.ac.uk/eva/). The authors should consider compressing the BAMs with CRAM and submitting them to the ENA too so the full chain of evidence is publicly available in a standard resource.

The variants going into the EVA will also mean the novel variants are assigned rs numbers and get properly integrated into dbSNP and Ensembl

Several places in the manuscript the chipname OmniZhongHua-8 is used as a name for the genotype chip used. If this is a custom chip design, that should be made clear. If it is a standard chip design, please use the illumina name for the chip

There are some typos/issues with the text

Manuscript page 1 Line 50: 83 of these 90 have not been sequenced by the 1000 Genomes Project

Should this be 7 of these 90 have not been sequenced by the 1000 Genomes Project

Manuscript page 3 line 40: All Samples used in this study where obtained from 1000GP

The correct statement here is that

All Samples used in this study were obtained from the 1000 Genomes Project cell line collection at the Coriell Cell line repository

Manuscript page 4 line 33 Tabel 3 should be Table 3

Manuscript page 5 line 61 It mentioned protocols.io without any link to the specific documents. This needs to be a link

Table 2. I am confused by the usage of the fraction of target covered statistic. This is general used for exome data and is not considered relevant for whole genome data as there are no targets to be covered.

From a style perspective, the pdfs for command lines are difficult to read, interpret and reuse. I would strongly advocate for the authors creating a public git repo or equivalent which gives text files and direct urls to link to the programs run and the versions of those programs used. This is much better from a reproducibility point of view.

Thanks

Laura Clarke

Level of Interest

Please indicate how interesting you found the manuscript: An article of importance in its field

Quality of Written English

Please indicate the quality of language in the manuscript: Acceptable

Declaration of Competing Interests

Please complete a declaration of competing interests, considering the following questions:

- Have you in the past five years received reimbursements, fees, funding, or salary from an organisation that may in any way gain or lose financially from the publication of this manuscript, either now or in the future?
- Do you hold any stocks or shares in an organisation that may in any way gain or lose financially from the publication of this manuscript, either now or in the future?
- Do you hold or are you currently applying for any patents relating to the content of the manuscript?

- Have you received reimbursements, fees, funding, or salary from an organization that holds or has applied for patents relating to the content of the manuscript?
- Do you have any other financial competing interests?
- Do you have any non-financial competing interests in relation to this paper?

If you can answer no to all of the above, write 'I declare that I have no competing interests' below. If your reply is yes to any, please give details below.

I declare that I have no competing interests

I agree to the open peer review policy of the journal. I understand that my name will be included on my report to the authors and, if the manuscript is accepted for publication, my named report including any attachments I upload will be posted on the website along with the authors' responses. I agree for my report to be made available under an Open Access Creative Commons CC-BY license (http://creativecommons.org/licenses/by/4.0/). I understand that any comments which I do not wish to be included in my named report can be included as confidential comments to the editors, which will not be published.

I agree to the open peer review policy of the journal
