# Supplementary material for: Deep whole-genome sequencing of 90 Han Chinese genomes
Source: Gigascience. 2017 Jul 31;6(9):1–7. doi: 10.1093/gigascience/gix067 (PMC5603764; doi:10.1093/gigascience/gix067)
Supplement: Reviewer-2-Report-(Original-Submission).pdf [file gix067_Reviewer-2-Report-(Original-Submission).pdf]

Reviewer Report

Title: Deep whole-genome sequencing of 90 Han Chinese genomes

Version: Original Submission **Date:** 12/2/2016

Reviewer name: Omer Gokcumen

Reviewer Comments to Author:

The study by Lan and Lin et al. provides high-depth sequencing data from 90 Chinese genomes. It is of interest to the human genomics community, especially those who are interested in rare variants, genomic structural variants, and de novo genome assemblies. The dataset is especially relevant since the authors sequenced multiple libraries with different insert sizes. This will allow researchers to construct integrative approaches for resolving complex regions of the genome and structural variants within them. Overall, the study is relevant to the field and the dataset provided here will be used by researchers with different interests. Having said these, before publication, some important points need to be addressed:1. It is my understanding that the samples that are sequenced here are the same as the Chinese samples sequenced by 1000 Genomes Project. This needs to be made clear in the abstract. Also, from various parts of the text read as if the authors collected the samples and/or secured the consent forms themselves. I am assuming that this is not the case and it should be clarified both throughout the main text, methods and abstract.2. In the abstract, and elsewhere in the text, there are implications that Chinese population is special with regards to the need for improved sequencing. This needs to be either toned down or explained better. For example, in the abstract it is stated that ".. data is not always sufficient for this purpose, especially for Chinese populations"... Why?3. The statements in the Background with regards to the accuracy of 1000 Genomes dataset is not accurately portrayed. To my knowledge, 1000 Genomes Project do not use standard variant calling tools on individual low-coverage genomes. Instead, they utilized custom variant calling pipelines integrating data from thousands of genomes. As such, the variant calls are highly accurate. It is, however, possible that some of the rare variants have been filtered out and there may be a good number of false-negatives within 1000 Genomes Phase 3 datasets. This paper would be able to catch those for the Chinese population. As such, the emphasis should be on calling rare variants that may have been missed by 1000 Genomes.4. Given that these samples have already been sequenced by 1000 Genomes, I am confused as to how additional regional characteristics would be captured by this study as it is claimed. This part should be rewritten as it is currently misleading.5. The strength of this dataset, in my opinion, is not necessarily identifying new single nucleotide variants, but to identify structural variants that may have been missed by 1000 Genomes. I would emphasize this section and add a paragraph describing this aspect of the dataset in the background.6. Even though this is a data report, I think it is required to have a comparison of the variant calls in this dataset with the 1000 Genomes variant calls from the same samples. This is important given that these samples have already been sequenced before and the novelty of this new dataset depends on how many novel variations that it will determine. As such, it would be helpful to see a basic comparison of the single nucleotide, insertion deletion and structural variants that were found in this study and 1000 Genomes for these samples.7. The language needs to be tightened. I understand

everything that the authors wrote. However, there are multiple instances of grammatical errors or unclear wording. As such, a careful reading and editing would be essential before publication. For example:- Abstract: line 26: has been primarily -> has primarily been- Abstract: line 49: low pass sequencing -> low coverage sequencing- Background: Backgrounds -> Background- Background Line 8: -> NGS -> Next generation sequencing (NGS)- Background Line 17: tens thousands -> tens of thousands

Level of Interest

Please indicate how interesting you found the manuscript: An article of importance in its field

Quality of Written English

Please indicate the quality of language in the manuscript: Needs some language corrections before being published

Declaration of Competing Interests

Please complete a declaration of competing interests, considering the following questions:

- Have you in the past five years received reimbursements, fees, funding, or salary from an organisation that may in any way gain or lose financially from the publication of this manuscript, either now or in the future?
- Do you hold any stocks or shares in an organisation that may in any way gain or lose financially from the publication of this manuscript, either now or in the future?
- Do you hold or are you currently applying for any patents relating to the content of the manuscript?
- Have you received reimbursements, fees, funding, or salary from an organization that holds or has applied for patents relating to the content of the manuscript?
- Do you have any other financial competing interests?
- Do you have any non-financial competing interests in relation to this paper?

If you can answer no to all of the above, write 'I declare that I have no competing interests' below. If your reply is yes to any, please give details below.

N.A

I agree to the open peer review policy of the journal. I understand that my name will be included on my report to the authors and, if the manuscript is accepted for publication, my named report including any attachments I upload will be posted on the website along with the authors' responses. I agree for my report to be made available under an Open Access Creative Commons CC-BY license (http://creativecommons.org/licenses/by/4.0/). I understand that any comments which I do not wish to

be included in my named report can be included as confidential comments to the editors, which will not be published.

I agree to the open peer review policy of the journal
