# Supplementary material for: Deep whole-genome sequencing of 90 Han Chinese genomes
Source: Gigascience. 2017 Jul 31;6(9):1–7. doi: 10.1093/gigascience/gix067 (PMC5603764; doi:10.1093/gigascience/gix067)
Supplement: Reviewer-3-Report-(Original-Submission).pdf [file gix067_Reviewer-3-Report-(Original-Submission).pdf]

Reviewer Report

Title: Deep whole-genome sequencing of 90 Han Chinese genomes

Version: Original Submission **Date:** 12/5/2016

Reviewer name: Avinash Ramu

Reviewer Comments to Author:

The authors make available an important resource, deep whole genome sequencing data of 90 Han Chinese samples. Since the majority of sequencing data generated so far has been on individuals of European descent this resource could potentially be of great use to other researchers working with genomic data from the Han Chinese population.

Please find my comments below.

1. In the background section the authors state "each sample from the 1000 Genomes Project has an average sequencing depth of only 5~7X, which is low that heterozygous variants are frequently incorrectly called, and false variants
are commonly introduced." Could the authors cite a manuscript to support this claim? If the authors are speculating here the tone of the sentence could be modified accordingly since the overall goals of the 1000 Genomes
Project were different from this project.

2. "The 1000 Genomes data is not suitable imputation panel for specifically
Chinese populations, due to regional genetic characteristics of the samples gathered for the panel." This sentence is a little unclear, could the authors elaborate? Since the samples sequenced by this project are the same as the 1000 Genomes wouldn't this data-set lead to similar results?

3. In the SNP/INDEL discovery section, the authors describe the number of common, low-frequency and rare
variants, could the authors mention the allele frequency cutoffs for these three classes?

4. The authors provide the annotation for the deletion SVs. Could the authors provide an average breakdown by SV type(
number of duplications, deletions, translocations etc)?

5. Have the authors compared their SNP/INDEL call-sets to the results from the 1000 Genomes Project. This could be
helpful in two ways a.) Discover any systematic errors if any in their variant-calling approach. b) Illustrate the power
of this analysis/data over the 1000 Genomes dataset.

6. The authors specify the advantages of this dataset for genotype imputation, I'm curious to hear if the authors
have taken a stab at this using this dataset for the same. Would 90 high-depth samples be sufficient for this? This analysis is of-course not critical to this data-note.

7. The authors include a genotype imputation step in their genotype calling steps, could the authors indicate which
reference panel was used for this step and mention if this choice might affect their genotype calling results?

8. Would it be possible to also include brief annotations of the SNVs and INDELs since the authors seem to do this
for the deletion SVs?

9. Could the authors indicate the samples from which the SV was called(perhaps in the INFO field) in the SV genotypes VCF file?

Minor grammatical errors

- "Background" instead of "Backgrounds" in the section title

- "Two stages were
involved in SV calling: the first phase is fast mapping, which using the BWA-SW to locate contigs in the reference genome. The second phase is the LASTZ aligner, to hold large gaps on the alignments."

- "High-depth sequencing of these samples
has supplied us with highly confident variant calls upon the samples"

Level of Interest

Please indicate how interesting you found the manuscript: An article whose findings are important to those with closely related research interests

Quality of Written English

Please indicate the quality of language in the manuscript: Acceptable

Declaration of Competing Interests

Please complete a declaration of competing interests, considering the following questions:

- Have you in the past five years received reimbursements, fees, funding, or salary from an organisation that may in any way gain or lose financially from the publication of this manuscript, either now or in the future?
- Do you hold any stocks or shares in an organisation that may in any way gain or lose financially from the publication of this manuscript, either now or in the future?
- Do you hold or are you currently applying for any patents relating to the content of the manuscript?
- Have you received reimbursements, fees, funding, or salary from an organization that holds or has applied for patents relating to the content of the manuscript?
- Do you have any other financial competing interests?
- Do you have any non-financial competing interests in relation to this paper?

If you can answer no to all of the above, write 'I declare that I have no competing interests' below. If your reply is yes to any, please give details below.

I declare that I have no competing interests

I agree to the open peer review policy of the journal. I understand that my name will be included on my report to the authors and, if the manuscript is accepted for publication, my named report including any attachments I upload will be posted on the website along with the authors' responses. I agree for my report to be made available under an Open Access Creative Commons CC-BY license (http://creativecommons.org/licenses/by/4.0/). I understand that any comments which I do not wish to be included in my named report can be included as confidential comments to the editors, which will not be published.

I agree to the open peer review policy of the journal
