# Supplementary material for: Deep whole-genome sequencing of 90 Han Chinese genomes
Source: Gigascience. 2017 Jul 31;6(9):1–7. doi: 10.1093/gigascience/gix067 (PMC5603764; doi:10.1093/gigascience/gix067)
Supplement: Reviewer-4-Report-(Original-Submission).pdf [file gix067_Reviewer-4-Report-(Original-Submission).pdf]

Reviewer Report

Title: Deep whole-genome sequencing of 90 Han Chinese genomes

Version: Original Submission **Date:** 12/6/2016

Reviewer name: Jason Flannick

Reviewer Comments to Author:

Sequencing additional populations is of importance for many genetic studies, particularly the GWAS/imputation approaches discussed in the introduction. The data produced by the current study, and the methods used to produce it, appears to be of high quality. However, I feel several points must be addressed to evaluate the extent to which this is an advance for the field.

(a) The authors state that the 90 sequenced samples are already a part of the 1000G project. Thus, their point in the introduction that "the 1000 Genomes data is not suitable imputation panel for specifically Chinese populations" did not make sense to me. How is this work addressing this limitation, if no new samples have been sequenced?

(b) If no new samples have been sequenced, then the main difference between the presented data and 1000G is the deep sequencing. Could the authors add a comparison as to how much is gained relative to 1000G as a result? Usually low-coverage sequencing is pretty good down to 0.5% frequency or so, below which imputation doesn't really work anyway. Thus the authors need to address how much is really gained from this study, ideally through a comparison to 1000G and with more detail of the types of variants that can now be studied/imputed better as a result.

(c) With imputation used as the motivating factor, the authors need to reference the HRC (http://biorxiv.org/content/early/2015/12/23/035170) and ideally reference how their work compares to it, rather than just 1000G. HRC is predominantly European but it should be mentioned.

(d) In general I'd like to see a few figures/tables showing the technical quality of the data. Do we have any confidence that the variants are accurate? How well did indel calling work? How about SVs? There are standard metrics for assessing this so it would be good to include some of them. In particular, seeing how these compared to other published efforts such as 1000G would be good.

Level of Interest

Please indicate how interesting you found the manuscript: An article of limited interest

Quality of Written English

Please indicate the quality of language in the manuscript: Acceptable

Declaration of Competing Interests

Please complete a declaration of competing interests, considering the following questions:

- Have you in the past five years received reimbursements, fees, funding, or salary from an organisation that may in any way gain or lose financially from the publication of this manuscript, either now or in the future?
- Do you hold any stocks or shares in an organisation that may in any way gain or lose financially from the publication of this manuscript, either now or in the future?
- Do you hold or are you currently applying for any patents relating to the content of the manuscript?
- Have you received reimbursements, fees, funding, or salary from an organization that holds or has applied for patents relating to the content of the manuscript?
- Do you have any other financial competing interests?
- Do you have any non-financial competing interests in relation to this paper?

If you can answer no to all of the above, write 'I declare that I have no competing interests' below. If your reply is yes to any, please give details below.

I declare that I have no competing interests

I agree to the open peer review policy of the journal. I understand that my name will be included on my report to the authors and, if the manuscript is accepted for publication, my named report including any attachments I upload will be posted on the website along with the authors' responses. I agree for my report to be made available under an Open Access Creative Commons CC-BY license (http://creativecommons.org/licenses/by/4.0/). I understand that any comments which I do not wish to be included in my named report can be included as confidential comments to the editors, which will not be published.

I agree to the open peer review policy of the journal
